# Haploinsufficiency in tumor predisposition syndromes: altered genomic transcription in morphologically normal cells heterozygous for *VHL* or *TSC* mutation

**DOI:** 10.18632/oncotarget.12192

**Published:** 2016-09-22

**Authors:** Suraj Peri, Elena Caretti, Rossella Tricarico, Karthik Devarajan, Mitchell Cheung, Eleonora Sementino, Craig W. Menges, Emmanuelle Nicolas, Lisa A. Vanderveer, Sharon Howard, Peggy Conrad, James A. Crowell, Kerry S. Campbell, Eric A. Ross, Andrew K. Godwin, Anthony T. Yeung, Margie L. Clapper, Robert G. Uzzo, Elizabeth P. Henske, Christopher J. Ricketts, Cathy D. Vocke, W. Marston Linehan, Joseph R. Testa, Alfonso Bellacosa, Levy Kopelovich, Alfred G. Knudson

**Affiliations:** ^1^ Department of Biostatistics and Bioinformatics, Fox Chase Cancer Center, Philadelphia, PA, USA; ^2^ Cancer Epigenetics, Fox Chase Cancer Center, Philadelphia, PA, USA; ^3^ Cancer Biology, Fox Chase Cancer Center, Philadelphia, PA, USA; ^4^ Cancer Prevention and Control, Fox Chase Cancer Center, Philadelphia, PA, USA; ^5^ Blood Cell Development and Function, Fox Chase Cancer Center, Philadelphia, PA, USA; ^6^ University of California San Francisco, San Francisco, CA, USA; ^7^ Developmental Therapeutics Program, Division of Cancer Treatment and Diagnosis, NCI, Rockville, MD, USA; ^8^ Department of Pathology and Laboratory Medicine, University of Kansas Medical Center, Kansas City, KS, USA; ^9^ Kidney Cancer Programs, Fox Chase Cancer Center, Philadelphia, PA, USA; ^10^ Brigham and Womens Hospital, Harvard Medical School, Boston, MA, NCI, Bethesda, MD, USA; ^11^ Urologic Oncology Branch, Center for Cancer Research, National Cancer Institute Bethesda, MD, USA; ^12^ Department of Medicine, Weill Cornell Medical College, New York, NY, USA

**Keywords:** VHL, TSC1, TSC2, transcriptomics, primary kidney epithelial cells

## Abstract

Tumor suppressor genes and their effector pathways have been identified for many dominantly heritable cancers, enabling efforts to intervene early in the course of disease. Our approach on the subject of early intervention was to investigate gene expression patterns of morphologically normal one-hit cells before they become hemizygous or homozygous for the inherited mutant gene which is usually required for tumor formation. Here, we studied histologically non-transformed renal epithelial cells from patients with inherited disorders that predispose to renal tumors, including von Hippel-Lindau (VHL) disease and Tuberous Sclerosis (TSC). As controls, we studied histologically normal cells from non-cancerous renal epithelium of patients with sporadic clear cell renal cell carcinoma (ccRCC). Gene expression analyses of *VHL*mut/wt or *TSC1*/*2*mut/wt versus wild-type (WT) cells revealed transcriptomic alterations previously implicated in the transition to precancerous renal lesions. For example, the gene expression changes in *VHL*mut/wt cells were consistent with activation of the hypoxia response, associated, in part, with the Warburg effect. Knockdown of any remaining *VHL* mRNA using shRNA induced secondary expression changes, such as activation of NF?B and interferon pathways, that are fundamentally important in the development of RCC. We posit that this is a general pattern of hereditary cancer predisposition, wherein haploinsufficiency for *VHL* or *TSC1*/2, or potentially other tumor susceptibility genes, is sufficient to promote development of early lesions, while cancer results from inactivation of the remaining normal allele. The gene expression changes identified here are related to the metabolic basis of renal cancer and may constitute suitable targets for early intervention.

## INTRODUCTION

The discovery of tumor suppressor genes (TSGs) and their pivotal role in the etiology of hereditary cancer presents the opportunity to study phenotypically normal-appearing, histologically non-transformed target tissues, for possible intervention [1]. This approach may also lead to early intervention of clinically-identifiable pre-neoplastic lesions such as polyposis of the colon and squamous or basal skin cancers, wherein hundreds of lesions appear before progression to carcinoma [2]. While efforts to prevent lesion formation have not been entirely successful, the use of anti-inflammatory drugs, as one such example, can result in decreased tumor formation [3]. We chose to study two forms of hereditary kidney cancer, i.e., von Hippel-Lindau disease (VHL) and Tuberous Sclerosis Complex (TSC), due to germline mutations of *VHL* or *TSC1*/2, respectively. Patients with VHL or TSC can develop hundreds of small benign renal lesions. In TSC, these lesions include angiomyolipomas, benign cysts, and renal cell carcinoma (RCC), all thought to be two-hit lesions, as is the case for angiofibromas (cortical tubers are still a matter of uncertainty) [4–6]. VHL is especially interesting because biallelic somatic inactivation of *VHL* occurs in the majority of sporadic (non-hereditary) RCCs [7, 8]. Therefore, our specific strategy has been to study histologically normal “one-hit” renal epithelial cells, i.e., heterozygous *VHL*mut/wt or *TSC1/2*mut/wt cells (referred to herein as *VHL*-single hit and *TSC1/2*-single hit cells) from affected kidneys of mutation carriers. Here, we report aberrant patterns of gene expression in non-transformed *VHL*- or *TSC1/2*-”one-hit” renal epithelial cells from patients with germline *VHL* or *TSC1/2* mutations. Importantly, the transcriptional changes that are differentially observed in these cells are suggestive of metabolic alterations that cause an altered energy production by the tricarboxylic acid (TCA) cycle and glycolysis. Specifically, the investigations reported here uncover early transcriptional changes on the path to RCC that might provide targets for interventions. Correspondingly, transcriptional alterations have also been described in one-hit cells from target tissues of patients with dominantly inherited susceptibility to colon or breast cancers [9–11].

The high rate of somatic *VHL* mutations in sporadic kidney cancers, particularly clear cell renal cell carcinomas (ccRCC) suggests that inactivation of the VHL protein plays a critical role in the initiation of RCC in the general population [7, 8]. As noted, the affected adult kidney from VHL patients typically contains hundreds of very small tumors that do not metastasize [12], wherein removal of the whole kidney is not necessary, providing a window for effective intervention before progression to metastatic cancer.

TSC is caused by inactivation of either *TSC1* or *TSC2*, leading to activation of the mammalian/mechanistic target of rapamycin complex 1 (mTORC1). The TSG activity of *TSC1* and *TSC2* depends upon the interaction of their respective protein products [13–15].

Consistent with earlier findings [16], transcriptomic profiles of morphologically normal, non-transformed (MNNT) kidney epithelial cells carrying germline mutations of *VHL* or *TSC1/2* are different from each other and from those of individuals not harboring a germline mutation (wild-type, *WT*) of these genes. The comparative expression profiling of kidney epithelial cells from *WT*, *VHL*-single hit, and *TSC1/2*-single hit individuals reported here may provide critical insights into renal oncogenesis, including deranged signaling pathways and altered intermediary metabolism. Importantly, the molecular alterations described here may serve as markers of early progression and aid in the development of novel intervention strategies.

## RESULTS

### Genome-wide transcriptome analysis

Our primary goal was to compare transcriptomes of MNNT renal epithelial cells harboring a mutation in one allele of *VHL* or *TSC1*/2, to monitor the earliest gene expression changes associated with one-hit inactivation of a given TSG [11, 16, 25]. To confirm that cultured cells from VHL patients retained their heterozygous status while grown *in* *vitro*, we performed *VHL* mutation analysis on five cultures. Four different monoallelic sequence variants were identified in four cultures: an in-frame deletion c.227_229delTCT was identified in cultures VHL-4 and VHL-5, whereas missense substitutions c.499C>T and c.473T>C were found in VHL-1 and VHL-6 cells, respectively (Supplemental Table 2; Supplemental Figure 1). Each change is pathogenic and previously reported in ccRCC or pheochromocytomas [26–29]. Additionally, a likely non-pathogenic missense substitution, c.21C>A, was identified in VHL-5. In each instance, the *VHL* mutation was heterozygous, with one allele being normal. In the fifth culture, no obvious *VHL* mutation was found, although clinical features of the corresponding patient were consistent with a diagnosis of VHL disorder. In each case, results *in vitro* conformed with those obtained upon admission of patients. Also, MNNT one-hit cells were obtained from six patients diagnosed with TSC1 or TSC2 based on distinctive clinical features, although mutational analysis is not available for this *TSC1/2* patient group.

**Figure 1:**
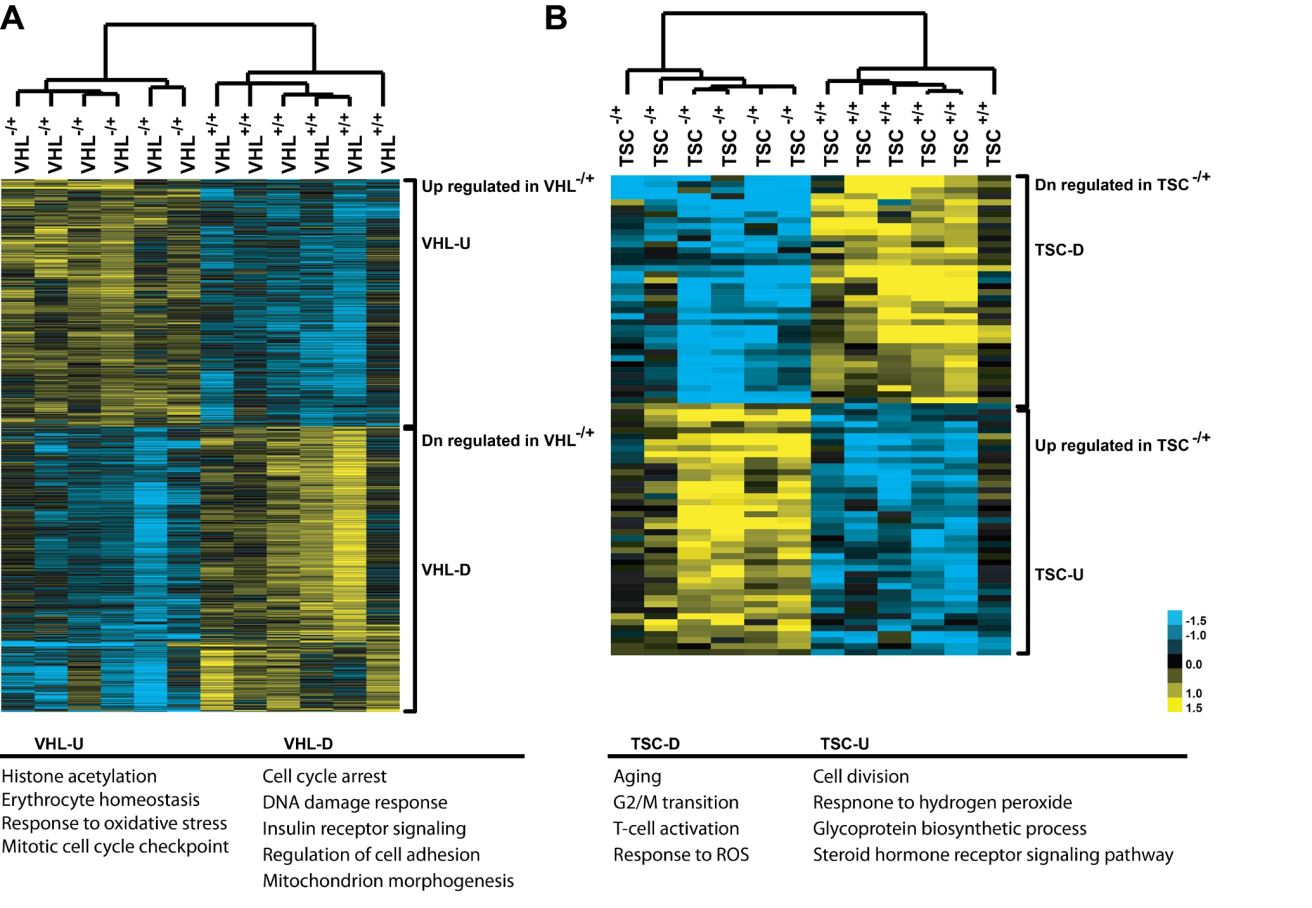
Gene expression patterns, as heatmap, between *VHL*^mut/wt^ and wild-type *(WT)* renal epithelial cells **(A)**, **and between TSC1/2mut/wt and WT renal epithelial cells (B)** **U, up-regulated; D, down-regulated**.

Next, we performed a global transcriptomic analysis on MNNT cells of *VHL*-single hit, *TSC1/2*-single hit, and *WT* individuals using Affymetrix U133plus2 chips that enabled better resolution of probesets [16]. Using a FDR cutoff of 20%, a total of 1,318 and 80 probe sets were differentially expressed between one-hit cells from VHL patients and WT controls (Supplemental Table 3), and between one-hit cells from TSC patients and WT controls (Supplemental Table 4), respectively. These probe sets correspond to a total of 1,036 differentially expressed genes for VHL cells and 62 differentially expressed genes for TSC cells. Figure 1 depicts a heatmap of genes differentially expressed between one-hit *VHL* or *TSC* and *WT* cells. We validated a fraction of the differentially expressed genes using real-time RT-PCR (Supplemental Tables 5, 6). Box plots depicting examples of differentially-expressed genes in *VHL* and *TSC* mutant cells are shown in Supplemental Figures 2 and 3, respectively.

Thus, comparative analyses of *VHL* one-hit (*VHL*mut/wt) *vs*. *WT* cells, and *TSC* one-hit (*TSC1/2*mut/wt) *vs*. *WT* cells revealed notable changes in the global gene expression, indicating that heterozygous germline mutations in *VHL* or *TSC1/2* do indeed affect the expression profiles of MNNT renal epithelial cells.

### Biological themes of *VHL* one-hit cells

To define biological themes, GO analysis was carried out on the 1,036 differentially expressed genes (571 down-regulated; 465 up-regulated) between *VHL*-single hit and *WT* cells, which revealed enrichment of several biological processes (Table 1). Genes up-regulated in *VHL*-single hit cells are involved in processes such as histone acetylation, oxidative stress and cell-redox homeostasis, including ubiquitin-dependent protein catabolic processes. Conversely, down-regulated genes involved DNA damage response, cell cycle arrest, mitochondrial morphogenesis, induction of apoptosis, and TGF-β signaling (Table 1).

**Table 1:** Gene Ontology (GO) categories (biological processes) enriched for both up and down-regulated genes in one-hit VHL cells

GOBPID	Term	Genes
GO:0043981	histone acetylation	MEAF6,PHF17,PHF16
GO:0007093	mitotic cell cycle checkpoint	ZWINT,RPS27L,CCNA2,CCNB1,BUB3
GO:0034101	erythrocyte homeostasis	FOXO3,ARNT,LYN,PRDX1,ACVR1B,SFXN1
GO:0030099	myeloid cell differentiation	FOXO3,ARNT,LYN,PSEN1,TGFBR2,CASP8,SCIN,ACVR1B,SFXN1,CDC42
GO:0043161	proteasomal ubiquitin-dependent protein catabolic process	ERLIN2,HSPA5,HSP90AB1,PSMA6,PSMA7,PSMD5,RAD23B,DERL1,CCNB1,BUB3
GO:0009060	aerobic respiration	IDH1,SDHB,UQCRH,CAT,SUCLG1
GO:0045454	cell redox homeostasis	PDIA6,DNAJC16,PDIA3,GSR,PRDX1,TXNDC12,SELT,PDIA4
GO:0042542	response to hydrogen peroxide	TXNIP,PRDX1,APTX,SLC8A1,CASP6,CAT
GO:0006979	response to oxidative stress	TXNIP,IDH1,ARNT,PRDX1,APTX,PSEN1,SLC8A1,TPM4,CASP6,ATRN,CAT
GO:0006511	ubiquitin-dependent protein catabolic process	UBE4B,ERLIN2,FBXO21,RNF144B,HSPA5,HSP90AB1,PSMA6,PSMA7,PSMD5,RAD23B,UBE2G1,DERL1,CCNB1,BUB3
GO:0055114	oxidation reduction	SLC25A13,KDM5B,HIBADH,EGLN3,COX15,CYP51A1,ALDH9A1,KDM1A,GLUD1,GSR,HADHA,HCCS,HGD,IDH1,MAOA,ALDH6A1,P4HA1,PRDX1,TXNDC12,RDH11,PCYOX1,KDM3B,OGFOD1,PECR,SDHB,SQLE,UQCRH,CYB5B,CAT
		
Downregulated Biological Processes		
GO:0070584	mitochondrion morphogenesis	DNM1L,COL4A3BP,OPA1
GO:0000045	autophagic vacuole assembly	ATG4B,C12orf44,ATG9A
GO:0008629	induction of apoptosis by intracellular signals	CDKN1A,C16orf5,PML,UACA,AEN,CUL4A
GO:0046626	regulation of insulin receptor signaling pathway	GRB14,PTPRF,RELA,TSC2
GO:0032570	response to progesterone stimulus	RELA,TGFB1,THBS1,FOSL1
GO:0008286	insulin receptor signaling pathway	AKT2,GRB14,PHIP,PTPRF,RELA,TSC2
GO:0043536	positive regulation of blood vessel endothelial cell migration	PDGFB,TGFB1,THBS1
GO:0007179	transforming growth factor beta receptor signaling pathway	LTBP2,MEN1,PDGFB,PML,TGFB1,TGFB1I1,THBS1,USP9Y,LTBP4
GO:0001952	regulation of cell-matrix adhesion	CDK6,RASA1,THBS1,TSC2
GO:0007162	negative regulation of cell adhesion	COL1A1,ARHGDIA,RASA1,TGFB1,THBS1
GO:0010810	regulation of cell-substrate adhesion	CDK6,COL1A1,RASA1,THBS1,TSC2
GO:0009411	response to UV	CDKN1A,MEN1,PML,ATR,TMEM161A,UACA,RELA
GO:0042770	DNA damage response, signal transduction	CDKN1A,C16orf5,PML,ATR,UACA,AEN,FBXO31,CEP63
GO:0006977	DNA damage response, signal transduction by p53 class mediator resulting in cell cycle arrest	CDKN1A,C16orf5,PML,ATR,AEN
GO:0007050	cell cycle arrest	CDKN1A,GAS2L1,MEN1,PML,MAPK12,TGFB1,THBS1,SESN2,CUL4A
GO:0031571	G1/S DNA damage checkpoint	CDKN1A,PML,FBXO31
GO:0031575	G1/S transition checkpoint	CDKN1A,PML,TGFB1,FBXO31
GO:0007064	mitotic sister chromatid cohesion	PDS5B,NIPBL
GO:0030036	actin cytoskeleton organization	SORBS3,FMNL2,DAAM1,FLNA,RND3,RHOG,PDGFB,CCDC88A,RAC2,RASA1,ROCK1,TSC2,CALD1,DIAPH3,PDLIM7,CYTH2,ARHGEF17

The enrichment analysis was done using GO-Stats package (Bioconductor). Categories were selected based on p-value cutoff < 0.01.

Importantly, several of the expression changes detected in *VHL* one-hit cells are consistent with the known biology of the VHL protein, including its role in the degradation of transcription factor hypoxia-inducible factor-1 (HIF1) under normoxic but not hypoxic conditions [8, 30].

**Figure 2:**
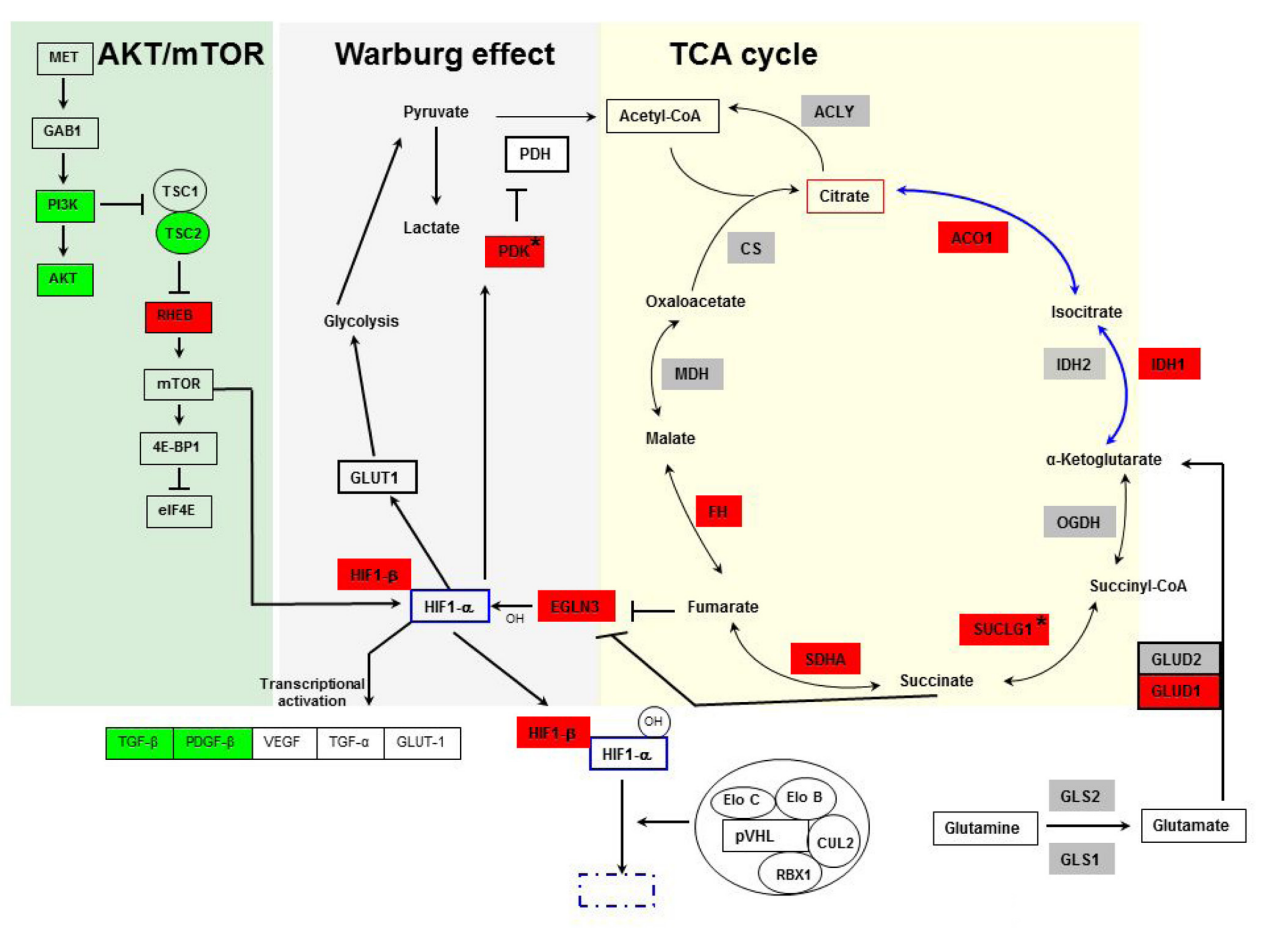
Pathways showing involvement of TCA cycle, Warburg effect, AKT-mTOR (yellow, gray and green background, respectively), and HIF in ***VHL***-single hit cells. Genes depicted in green indicate down-regulation, and red indicate up-regulation in *VHL*-single hit cells *vs*. *WT* cells. Genes not colored and shown in gray color are not differentially expressed. *VHL*-single hit cells show partial activation of Warburg effect, whereby entry of pyruvate into TCA cycle is restricted due to activation of PDK, whereas other arm of the Warburg effect through HIF activation of glucose transporter and glycolysis is not active. mTOR-mediated transcriptional activation of HIF is active. *, genes experimentally validated.

Using pathway analyses and data mining to evaluate the transcriptome of *VHL*-single hit cells, we found gene alterations involved in signal transduction, glycolysis and TCA cycle (Figure 2; Supplemental Figure 4). Signaling defects relevant to RCC involved MET, PI3K/AKT and HIF1α pathways. In *VHL*-single hit cells *PIK3R2*, *AKT2* and *TSC2* were down-regulated, whereas *RHEB*, encoding an activator of mTOR, was up-regulated. HIF1-mediated hypoxia signaling is critical in both sporadic and VHL-associated RCC subtypes. Although the *HIF1*α (*HIF1A*) and *EPAS1* (*HIF2A*) genes were not differentially expressed, we found that the *HIF1β (ARNT)* gene is up-regulated in *VHL* one-hit cells. When stabilized either under hypoxia or pseudo-hypoxic conditions, together with HIF1β, HIF1α induces downstream target genes, e.g., *VEGF*, *TGFA*, *TGFB*, *PDGFB* and *GLUT1*. We also found that genes encoding TGFβ and PDGFβ, which are involved in angiogenesis and induction of growth hormone signaling, are down-regulated (Figure 2; Supplemental Figure 4); this may be because additional transcription factors, such as AP-1, are necessary for cooperation with HIF and induction of VEGF [31].

Importantly, we found evidence for partial activation of the HIF pathway in *VHL*^mut/wt^ cells, because its transcriptional target, *EGL-Nine homolog 3* (*EGLN3/PHD3*) [32] is significantly overexpressed. EGLN3 and its family members serve as critical cellular oxygen sensors encoding proline hydroxylases involved in HIF1α degradation [32]. Under hypoxic conditions, increased expression of EGLN3 is necessary for survival and G1 to S transition of tumor cells [33], suggesting that in *VHL*-single hit cells a condition of modified steady state and feedback regulation exists that favors RCC development. Reduced activity of pVHL in *VHL*-single hit cells might lead to partial degradation of hydroxylated HIF1α, permitting limited stabilization of HIF1α leading to activation of its downstream targets. Consistent with this possibility, we observed overexpression of the pyruvate dehydrogenase kinase gene (*PDK*), several TCA cycle genes, e.g., *SDHB*-encoding succinate dehydrogenase, and genes encoding components of the isocitrate dehydrogenase, reductive glutamine pathway, e.g., *GLUD1*, *IDH1* and *ACO1*. Notably, PDK inhibits pyruvate dehydrogenase and thereby blocks import of pyruvate into the TCA cycle [34, 35] (Figure 2).

**Figure 3:**
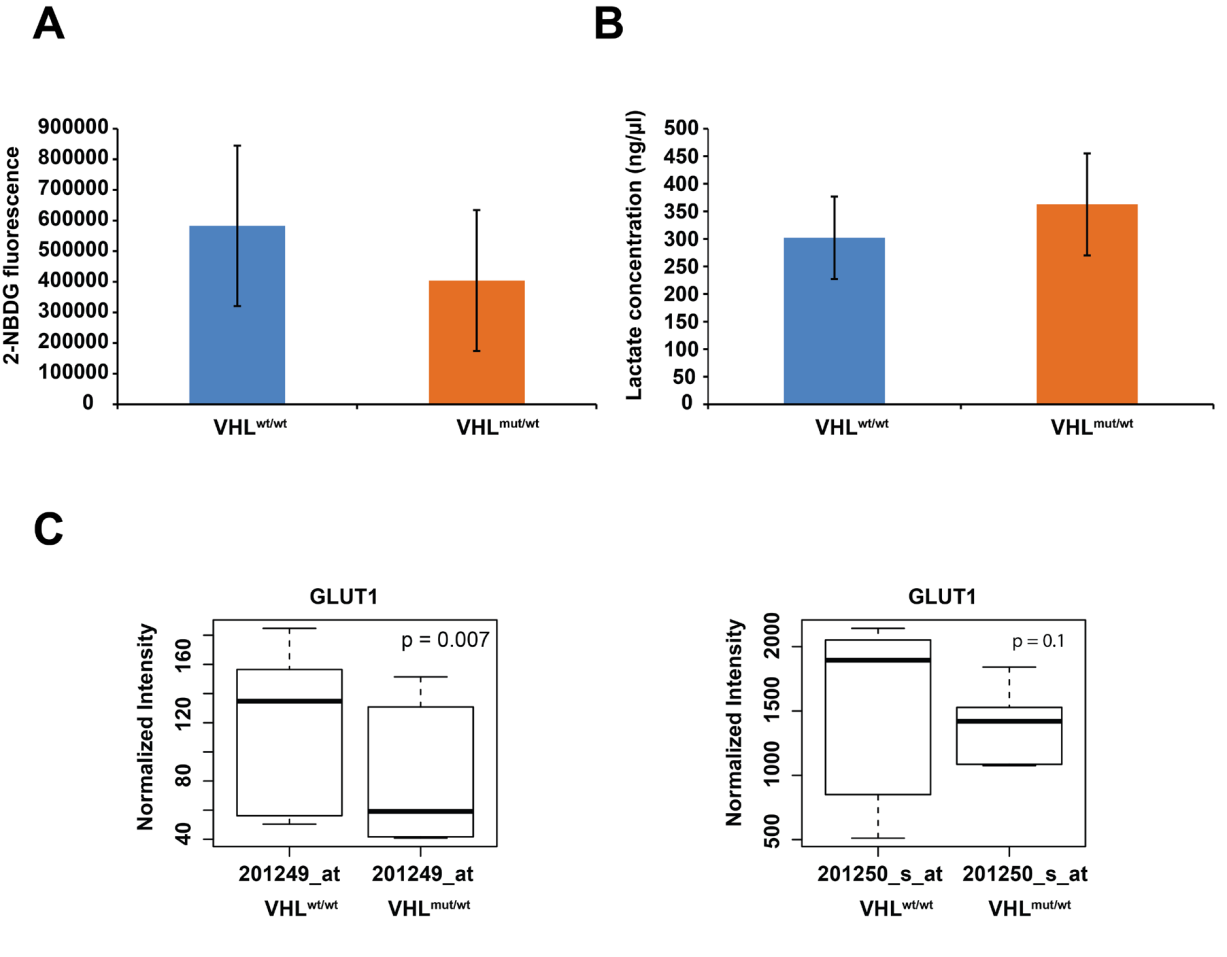
**A**. **Summary of averaged 2-NBDG fluorescence, indicating glucose uptake for 5 WT and 5 VHL-single hit cultures. B. Summary of averaged lactate concentration for 5 WT and 5 VHL-single hit cultures**. **C**. Box plots of differential *GLUT1* expression between VHL^mut/wt^ *vs*. *WT* cells, as determined with indicated Affymetrix probes.

To verify the contention of partial activation of HIF1α targets in *VHL* one-hit cells, we determined if these cells show altered glucose uptake and lactate production. While we observed decreased glucose uptake in comparison to *WT* cells (Figure 3A), lactate production was increased (Figure 3B). These results are corroborated by our finding of reduced *GLUT1* expression (Figure 3C), resulting in reduced glucose uptake. Overexpression of *PDK1* (Figure 2) would inhibit decarboxylation of pyruvate mediated by pyruvate dehydrogenase, leading to increased lactate in the cytosol. These results suggest that in *VHL*-single hit cells, increased PDK expression and lactate production, but not glucose consumption, represents the initial events of the “Warburg effect”, whereas in the classic Warburg effect of two-hit *VHL*mut/mut cancer cells and, indeed, in essentially most cancers, both lactate production and glucose consumption are elevated.

Besides alterations in the expression of TCA cycle-related genes, we observed overexpression of *PGK1*, encoding phosphoglycerate kinase 1, a critical glycolytic enzyme that catalyzes conversion of 1,3-diphosphoglycerate to 3-phosphoglycerate. PGK1 also acts as an ‘angiogenic switch’ whereby overexpression of PGK1 reduces secretion of VEGF1 and IL8 through increased angiostatin, whereas, at metastatic sites, high levels of CXCL12 negatively regulate PGK1 expression, thereby enhancing angiogenic response for metastatic growth [36]. Interestingly, while PGK1 is overexpressed in *VHL*-single hit cells, CXCL12 is nearly 4-fold up-regulated (log_2_ 1.9) in VHL-depleted cells (*VHL*^knock-down^; see below), suggesting the importance of VHL for its regulation.

**Figure 4:**
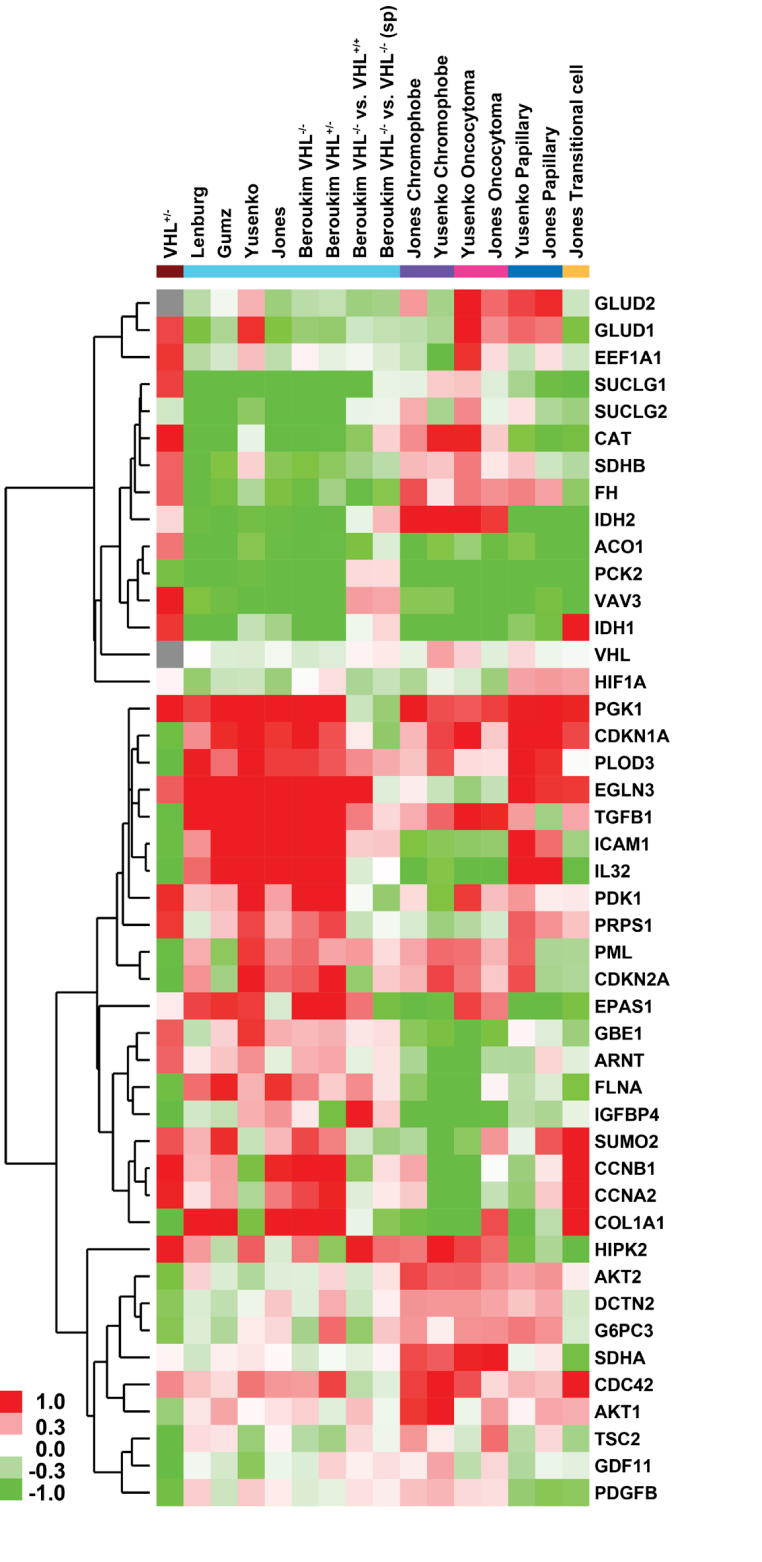
Heatmap of glycolysis, TCA cycle, HIF and AKT pathway genes depicting fold-changes in ***VHL***-single hit cells and different kidney malignancies compared to their respective normal kidney epithelium (from studies in Supplemental Table 1). ccRCC, clear cell RCC; pRCC, papillary RCC; trans-cRCC, transitional cell RCC. Datasets normalized using RMA and LIMMA were used to calculate fold -changes (no p-value cutoff was enforced). Green and red color in heatmap indicates down- and up-regulation in RCC or VHL^mut/wt^ cells, whereas gray indicates no fold change. Brown indicates *VHL*-single hit cells; cyan indicates studies of ccRCC, including comparisons based on *VHL* mutation status, a) among sporadic tumors with loss of two copies *vs*. one copy of *VHL* loss; b) *VHL* two-hit sporadic tumor *vs*. familial *VHL* loss cases; purple indicates chromophobe; magenta indicates oncocytoma; blue indicates papillary; and beige indicates transitional-cell RCC. Genes in matrix were hierarchically clustered (average linkage with uncentered Pearson correlation).

To delineate a common theme of differential gene expression patterns across published transcriptomic profiling studies of RCC subtypes and our non-transformed *VHL*-single hit kidney epithelial cells, we compared over- and under-expression of 45 genes involved in the Warburg effect, glycolysis, TCA cycle, and AKT/mTOR signaling (Figure 4). This analysis revealed that *VHL*-single hit cells exhibit a distinct gene expression profile, different from ccRCC tumor and WT cells. However, a similar over-expression pattern is observed for genes such as *GLUD1, PGK1, EGLN3, PDK1, PRPS1, PCK2, GBE1, HIF1β, SUMO2, CCNB1, CCNA2, HIPK2* and *CDC42* (Figure 4). Similar results were observed when we compiled a list of 268 genes that are known to be involved in these pathways (including HIF1α targets); of these, 189 genes can be compared across published studies in a heatmap (Supplemental Figure 5).

### Transcriptomic profile of *VHL*-single hit^VHL knock-down^ cells

If the expression changes in *VHL*-single hit cells represent an initial step during renal tumorigenesis, we hypothesized that rendering these cells *VHL*-null (i.e., two-hit inactivation) would further perturb their transcriptome and influence other causal mechanisms of transformation, as in ccRCC. We showed previously, through meta-analysis of multiple cancer datasets, that in a majority of ccRCC samples, NFκB is constitutively active, and that its key regulators and targets are uniformly up-regulated [37]. We also reported an enriched IFN signature in ccRCC [37]. Both NFκB and IFN gene sets are overexpressed in ccRCC samples where *VHL* is biallelically inactivated, but not in cells having functional VHL [37].

Thus, we knocked down the remaining *VHL* mRNA by infection with lentivirus-expressing *VHL* shRNA. The resulting cells, designated as *VHL*-single hit^VHLknock-down^, were profiled using expression arrays and compared to lentiviral vector-infected cells, designated *VHL*-single hit^pLKOcontrol^. Using a FDR cutoff of 20%, a total of 3,090 probe sets, corresponding to 2,018 genes, were differentially expressed between *VHL*-single hit^VHLknock-down^ and *VHL*-single hit^pLKOcontrol^ cells (Supplemental Table 7). Loss of any remaining *VHL* mRNA resulted in overexpression of 14 NFκB target genes. Of interest, *IFNB1, BCL2A1* and *IKBKE*, each a direct target of NFκB and mediator of both canonical and non-canonical IFN signaling and innate immunity, were overexpressed upon loss of expression of the second copy of *VHL* (Supplemental Table 7).

**Figure 5:**
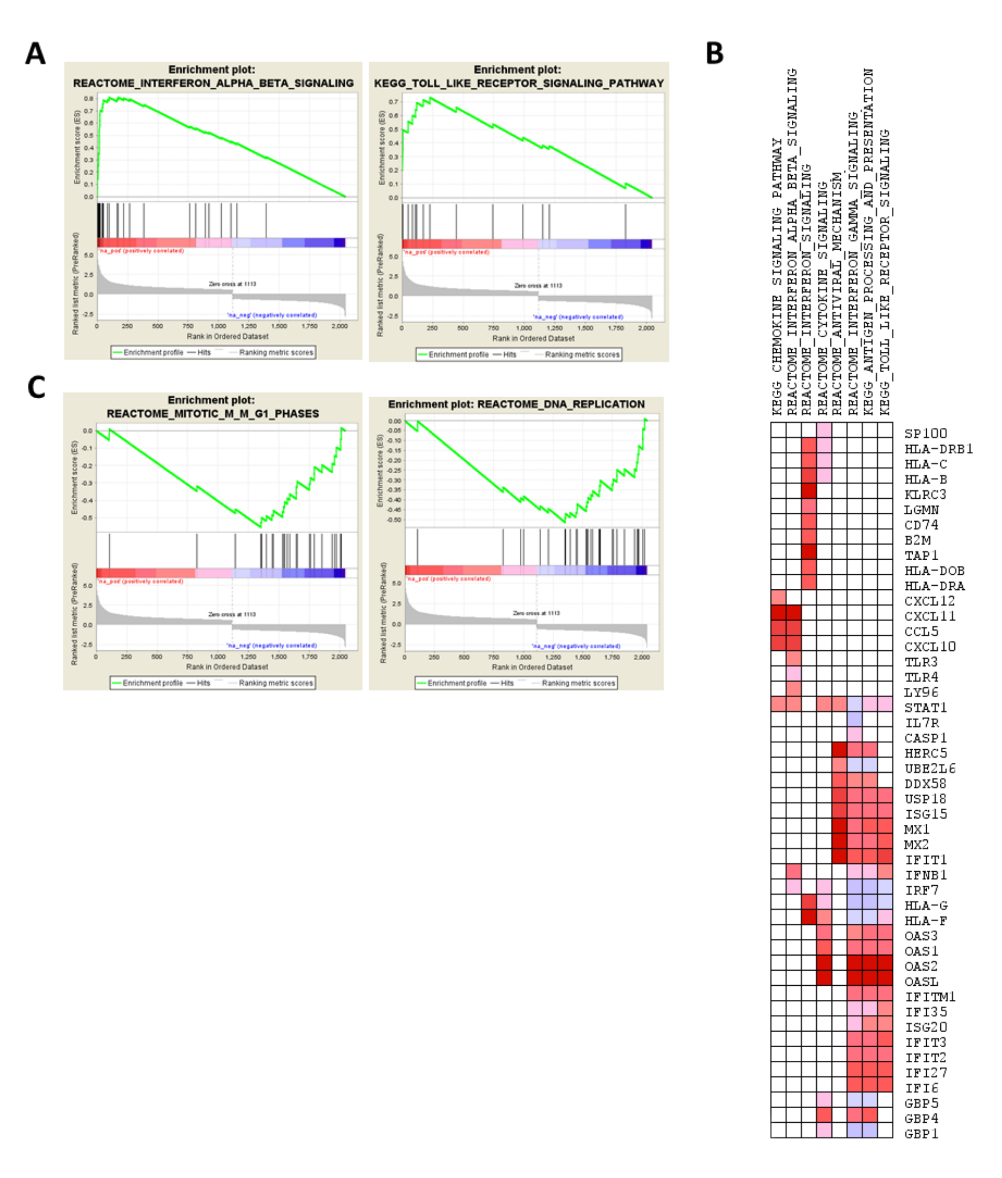
Enrichment analysis of the transcriptomic profile of ***VHL***-null cells, i.e., comparison of *VHL*-single hit^VHL knockdown^ *vs*. *VHL*-single hit^pLKOcontrol^. **A**. Enrichment plot for IFNB and TLR pathways. **B**. Pathways enriched for down-regulated genes (mitosis and DNA replication pathways in Reactome database). **C**. Leading edge analysis of up-regulated genes contributing to enrichment of pathway sets curated for interferon and anti-viral signaling pathways, depicted as heatmap (red and blue color indicate over- and underexpression of genes in *VHL*-single hit^VHL knockdown^ cells).

Analysis of GO categories (Supplemental Table 8) using GOstats package revealed enrichment of biological processes, including innate immunity and anti-viral signaling pathways. Similarly, enrichment analysis of pathways, using GSEA, indicated that interferon signaling, toll-like receptor (TLR) signaling (Figure 5A-5B) are enriched for overexpressed genes, whereas genes involved in cell cycle and replication are enriched for underexpressed genes in one-hit VHL^knock-down^ cells (Figure 5C; Supplemental Table 9).

### Transcriptomic profiles of TSC mutant cells

Unlike *VHL*-single hit cells, only 62 genes (corresponding to 80 differentially expressed probesets) were differentially expressed in *TSC1*/2^mut/wt^ cells, suggesting that monoallelic inactivation of *TSC1* or *TSC2* leads to a relatively small perturbation of the gene expression profile. GO analysis of differentially expressed genes (30 down-regulated; 32 up-regulated) revealed that genes involved in response to hydrogen peroxide and cell division are significantly up-regulated, whereas genes involved in T cell activation, regulation of G2/M transition, and reactive oxygen species are down-regulated (Table 2). Furthermore, compared to WT cells, *TSC1*/2-single hit cells showed down-regulation of multiple genes encoding endothelial markers and cell adhesion molecules, while several oncogenes, including *ERBB4* and *VAV3*, were up-regulated (Supplemental Table 4). Dramatic loss (~25-fold) of expression of *AQ-1*, encoding water channel aquaporin, with preferential localization in the renal proximal convoluted tubules and the descending thin limb of the loop of Henle, was observed in *TSC1*/2-single hit cells. AQ-1 is considered a differentiation marker of proximal renal tubular cells and its down-regulation is associated with loss of the differentiated phenotype and poor prognosis in RCC [38]. Thus, these data provide additional evidence that some features of the clinical phenotype are already present in one-hit cells, in this case heterozygous *TSC1*/2^mut/wt^ cells. Down-regulation of genes encoding endothelial markers, e.g., *VCAM1*, *MCAM* and *THBD*, was also observed. The *WT1* gene, a TSG often mutated in pediatric Wilms’ tumor, was also down-regulated in *TSC1*/2-one-hit cells (Supplemental Table 4). WT1 negatively regulates the cell cycle and induces apoptosis through transcriptional regulation of the pro-apoptotic factor BCL2. WT1 also controls the mesenchymal-epithelial transition during renal development [39]. Altered expression of several of these genes was confirmed by real-time RT-PCR analysis (Supplemental Table 6).

**Table 2:** Gene Ontology (GO) categories (biological processes) enriched for both up and down-regulated genes in one-hit TSC1/2 cells

GOBPID	Term	a3
GO:0010986	positive regulation of lipoprotein particle clearance	LIPG
GO:0060074	synapse maturation	ERBB4
GO:0051301	cell division	CAT,CCNA2,CCNG2,CDK1
GO:0007095	mitotic cell cycle G2/M transition DNA damage checkpoint	CCNA2
GO:0043551	regulation of phosphoinositide 3-kinase activity	VAV3
GO:0050847	progesterone receptor signaling pathway	UBR5
GO:0030518	steroid hormone receptor signaling pathway	UBR5,MED14
GO:0051973	positive regulation of telomerase activity	PARM1
GO:0042542	response to hydrogen peroxide	ERBB4,CAT
GO:0009101	glycoprotein biosynthetic process	MGAT4A,ST8SIA4,CHST9
		
Downregulated biological processes		
GO:0007568	aging	CDKN2A,CRYAB,SERPINE1
GO:0001300	chronological cell aging	SERPINE1
GO:0002691	regulation of cellular extravasation	ICAM1
GO:0022614	membrane to membrane docking	ICAM1,VCAM1
GO:0042110	T cell activation	CDKN2A,ICAM1,VCAM1
GO:0033079	immature T cell proliferation	CDKN2A
GO:0032836	glomerular basement membrane development	WT1
GO:0010389	regulation of G2/M transition of mitotic cell cycle	CDKN2A
GO:0031641	regulation of myelination	CDH2
GO:0000302	response to reactive oxygen species	CRYAB,SERPINE1

The enrichment analysis was done using GO-Stats package (Bioconductor). Categories were selected based on p-value cutoff < 0.01.

## DISCUSSION

In this report, we identified altered gene expression patterns in cultured, MNNT one-hit renal epithelial cells of patients with VHL or TSC as compared to WT kidney epithelial cells. VHL and TSC are two of the ~10 phakomatoses that are characterized by dominant inheritance and scattered heterozygous precancerous lesions. These lesions are examples of haploinsufficiency for TSG mutations that may cause malignancy upon transition to the homozygous state [40].

Expression of genes involved in processes associated with the Warburg effect of aerobic glycolysis was altered in normal appearing *VHL*-single hit cells. However, this hypoxic response in *VHL*-single hit cells appears to be incomplete, since HIF1α activity was not associated with overexpression of some of its canonical downstream targets, such as *VEGF* and *GLUT1*. Yet overexpression of *EGLN3,* a transcriptional target of HIF1α [41, 42], does indicate that *VHL*-single hit cells are under hypoxic stress [41]. EGLN3 is involved in the G1>S transition in carcinoma cells [33] wherein its up-regulation is a direct response to hypoxia as a means for survival while under stress [33, 43]. Importantly, we did find increased expression HIF1β that is needed in order to confer stability and permit DNA binding of HIF1α, confirming our contention of the nature of events occurring early on in one-hit cells.

In further support of our contention of incomplete hypoxic response, while changes in expression levels of both HIF1α, HIF2α are not observed in our *in* *vitro* model, an *in vivo* mouse model of *Vhl* one-hit type 2B mutation (R167Q) shows low basal level expression in both these genes similar to cells with intact VHL, suggesting the pVHL in *VHL* one-hit cells does not stabilize HIF proteins. Interestingly, mice with *Vhl* one-hit type 2B mutation develop renal cysts and fail to progress to renal adenocarcinoma, suggesting *VHL* mutation alone is not sufficient for transformation [44].

In ccRCC, stabilization of HIF1*α* upon loss of VHL leads to activation of the Warburg effect through increased glucose uptake and lactate production, including restricted entry of pyruvate into mitochondria through activation of PDK1, a HIF1α target [34, 35]. Shunting of pyruvate away from the TCA cycle reduces levels of acetyl-coA, a glucose-derived carbon source for lipid biogenesis [45]. Interestingly, *in vitro* studies indicate that cancer cells supplement deficiency of acetyl-coA for lipid biogenesis through reductive glutamine metabolism mediated by cytoplasmic and/or mitochondrial IDH1 and IDH2, respectively [43, 44].

In *VHL*-single hit cells, we observed overexpression of *PDK1*, suggesting that the resulting inactivation of PDH would shunt pyruvate from mitochondria to cytosol with subsequent conversion of pyruvate to lactate. Indeed, we demonstrated increased lactate production in *VHL-*single hit cells (Figure 3B).

However, expression of GLUT1, a critical HIF1α target involved in the Warburg effect, is not elevated in *VHL* one-hit cells. In keeping with reduced expression of GLUT1, glucose uptake is lower in *VHL*^mut/wt^ *vs*. *WT* cells (Figure 3A). Thus, increased lactate production, but not glucose uptake, conforms with our observation that *VHL*^mut/wt^ cells are transcriptionally different from *WT* and ccRCC cells, and that they presumably represent the earliest phases of malignant transformation, i.e., cancer initiation (1). In this context, it should be noted that contrary to tumor cells, low glucose content in non-tumor cell lines has been shown to increase the stability of HIF1α [46].

Interestingly, we also observed that TCA cycle genes such as those encoding succinate-CoA ligase (*SUCLG1*), succinate dehydrogenase complex (*SDHB*), and fumarate hydratase (*FH*) are over-expressed in *VHL*-single hit cells. This suggests that, in these cells, like most non-malignant cells, the TCA cycle progresses largely towards acetyl-CoA in the oxidative state. Yet, in *VHL*-single hit cells we also observed overexpression of genes encoding cytosolic ACO1 and IDH1 - critical components of reductive glutamine metabolism - again suggesting that these cells, in addition to effective forward progression of the TCA cycle, have also activated an oncogenic metabolic shift that is seen in RCC, including a partial reversal of the TCA cycle.

To determine the effect of loss of the remaining functional allele of *VHL* in *VHL*-single hit cells, we depleted the remaining *VHL* mRNA using *VHL* shRNA lentiviral infection. Transcriptomic profiles of these *VHL* knockdown cells revealed overexpression of several direct targets/effectors of NFκB and IFN, including *IRF7*, *STAT1*, *IFNB1* and *IKBKE*. Consistent with this, we recently reported overexpression of NFκB and IFN signaling in *VHL*-null ccRCC cells [37]. This is also consistent with studies showing that loss of VHL leads to activation of NFκB through CARD9, an agonist for NFκB [47].

Fewer changes were observed in TSC-mutant cells, indicating that heterozygosity for *TSC1/2* has a lesser impact on transcription than monoallelic *VHL* mutations. TSC1 and TSC2 are negative regulators of AKT/mTOR signaling, and inhibitors of mTOR are efficacious in TSC patients. AKT/mTOR signaling is activated in many human cancers, including RCC [48]. Thus, it is possible that *TSC1/2* haploinsufficiency is sufficient to shift cells toward the initiated state, which would further sensitize them to factors associated with malignant conversion.

In conclusion, analyses of primary cell cultures from kidneys of individuals with or without *VHL* or *TSC1/2* mutations indicate that heterozygosity for TSG mutations is associated with detectable changes in gene expression that can be characterized as the initiated state in otherwise histologically normal cells. Many of these changes are consistent with the biology of homozygously-mutant RCC cells, thereby supporting the idea that gene alterations in one-hit cells represent relevant risk biomarkers and potential targets for therapeutic or preventive agents. We also note that since most non-hereditary ccRCCs are mutant for *VHL*, these results may be relevant to the management of this much larger group of renal cancers. Agents that reverse these pathways in VHL or TSC might be useful in the earliest stage of oncogenesis, perhaps even in preventing loss of the wild-type TSG allele in predisposed individuals.

## MATERIALS AND METHODS

### Subject accrual and specimens

All VHL, TSC and control (WT) subjects were recruited with the approval of the Fox Chase Cancer Center (FCCC) or National Cancer Institute (NCI) Institutional Review Board, and were chosen irrespective of gender, race and age. Specimens and accompanying information were de-identified. Individuals treated previously with chemotherapy or radiation were ineligible. Kidney biopsies were collected during surgery performed at NCI (VHL patients) and hospitals throughout the USA (TSC patients). As control tissues, we used normal renal epithelium adjacent to sporadic renal tumors from patients who underwent nephrectomy; these were designated WT, due to the absence of *VHL* or *TSC1/2* mutations. In total, kidney epithelium was accrued from 6 *VHL* patients, 6 *TSC1/2* mutation carriers, and 6 *WT* controls.

### Establishment of primary kidney epithelial cell cultures

Preparation of early passage (2-5) renal epithelial cultures was as described [16]. They grew robustly in culture (doubling time, 48 h) and did not show overt signs of transformation; all cultures underwent senescence by passages 7-10. These renal epithelial strains are defined here as *VHL*-single hit (or *VHL* mutant), *TSC*-single hit (*TSC* mutant) and control (*WT*) cells. Cultures from mutation carriers were phenotypically indistinguishable from those derived from *WT* controls and did not differ with regard to proliferation rate or level of apoptosis [16].

### *VHL* mutational analysis

To confirm that the primary cultures from VHL patients retained their “one-hit” status *in vitro*, we performed a *VHL* mutation analysis on five of them. Genomic DNA was extracted using the Gentra Puregene Tissue Kit (Qiagen), per the manufacturer's protocol. The complete coding sequence and flanking exon-intron borders of *VHL* were investigated by direct sequencing. PCR reactions were performed using PFU Turbo (Agilent) following cycling program: 95°C for 2 min; 35 cycles of (95°C 30 sec, 65°C 30 sec, 72°C 1 min); 72°C for 10 min. PCR products were gel-purified and sequenced using forward and reverse primers: preT7, (GGGAGGTCTATATAAGCAGa) and T3 (ATTAACCCTCACTAAAGGGA), respectively. Mutation nomenclature is in accordance with HGVS (http://www.hgvs.org/mutnomen/) recommendations. *VHL* cDNA sequences (GenBank accession #NM_000551) are shown with A of the ATG translation-initiation codon numbered as +1.

### VHL down-regulation in one-hit kidney epithelial cells

Short hairpin RNA (shRNA) lentiviruses targeting human *VHL* (named sh*VHL* B2 and sh*VHL* B4), with the sequences CAATGTTGACGGACAGCCTAT and TAGGATTGACATTCTACAGTT, were from Thermo Scientific OpenBio-Systems. Control lentivirus pLKO and shVHL B2 and shVHL B4 lentiviral constructs and appropriate packaging plasmids were transfected into 95% confluent 293T cells with the ViraPower™ Lentiviral Expression System (Invitrogen, Life Technologies). Supernatants were harvested 48 h after transfection and filtered through 0.45-μm pore filters (Millipore). Five primary VHL renal epithelial cell cultures were transduced with either control lentivirus (pLKO) or lentiviruses expressing shVHL B2 or shVHL B4 in the presence of 6 μg/ml polybrene (Santa Cruz) and analyzed 5-7 d after infection. Resulting strains are referred to as one-hit VHL^pLKO control^ and one-hit VHL^knock-down^, respectively.

### Immunoblotting

Protein extracts were prepared from cultured cells transiently transduced with shVHL B2, shVHL B4 or control shRNA, using RIPA buffer (50 mM Tris HCl pH 7.4, 150 mM NaCl, 1% sodium deoxycholate, 1% Triton X-100, 0.1% SDS, 10 mM NaF, and 1 mM each of sodium pyrophosphate, sodium orthovanadate, dithiothreitol, and EDTA), plus protease inhibitors. Lysates were fractionated by SDS-PAGE and transferred to PVDF membranes (Millipore). Membranes were blocked in 4% nonfat dry milk in TPBST and incubated with anti-VHL antibody from GeneTex, 1/1000 dilution in 4% nonfat dry milk in TPBST. Anti-β-actin (Sigma) was used as a loading control. Detection was performed using enhanced chemiluminescence (Amersham).

### RNA extraction and amplification

Total RNA was extracted from cultured cells using a guanidinium isothiocyanate-based buffer containing β-mercaptoethanol and acid phenol [11]. Amplification of total RNAs was with the one-cycle Ovation™ biotin system (NuGEN) [17].

### Hybridization and microarray analysis

For each sample, 2.2 μg of ssDNA, labeled and fragmented using a NuGEN kit, was hybridized to Affymetrix arrays (Human U133 plus 2.0) as described [17]. After washing and staining with biotinylated antibody and streptavidin-phycoerythrin, arrays were scanned with an Affymetrix GeneChip Scanner 3000 for data acquisition.

### Real-time reverse transcriptase-PCR (RT-PCR) validation of microarray data

Validation of microarray findings was performed by real-time RT-PCR on microfluidic cards, TaqMan Low Density Arrays (LDA; Applied Biosystems). A 48-gene custom array (47 candidate biomarkers and 1 housekeeping gene, *GAPDH*) was from Applied Biosystems. This panel was tested for all samples in quadruplicate to ensure accuracy and reproducibility.

Data were obtained in the form of threshold-cycle number (C_t_) for each candidate biomarker identified and *GAPDH* for each genotype (*WT, VHL*-single hit, *TSC1/2*-single hit). For each gene, C_t_ values were normalized to *GAPDH*, and the corresponding C_t_ values obtained for each genotype. Relative quantitation was computed using the Comparative C_t_ method (Applied Biosystems Reference Manual, Bulletin #2) between *VHL* or *TSC1/2* mutants and *WT* primary cell RNAs. Relative quantitation is the ratio of normalized amounts of mRNA target for *VHL*, *TSC1/2* and *WT* cells, and was computed as 2^(-C_t_) where ∆C_t_ is the difference between mean C_t_ values for *VHL* or *TSC1/2* mutant and mean ∆C_t_ values for *WT* RNAs.

### Statistical analysis of microarray data

Primary cultures were analyzed for each genotype: *VHL* (*VHL*mut/wt), *TSC* (*TSC1*mut/wt or *TSC2*mut/wt), and *WT* (both *VHL*wt^/wt^ and *TSC*wt^/wt^), using six biological replicates per experimental condition (total: 18 samples). For each sample, probe-level data in the form of raw signal intensities from Affymetrix arrays were preprocessed using the Robust Multi-chip Average (RMA) method [18].

Linear Models for Microarray Data (LIMMA) was used for class comparisons [19–21]. All comparisons were two-sided. The Benjamini-Hochberg method [22] was applied to control False Discovery Rate (FDR). Differentially expressed genes were identified based on statistical significance (FDR < = 20%) and biological significance (up- or down-regulated ≥ 2-fold) and validated using RT-PCR, with the analysis comparing five paired VHL shRNA- and control-infected primary *VHL*-single hit strains.

### Mining for functional categories and pathways

Gene ontology (GO) functional categories, enriched in over- and under-expressed genes, were identified using conditional hyper-geometric tests in the GOstats package [23]. A p-value cutoff of 0.01 was used in selecting GO terms. Gene networks were generated using Ingenuity Pathway Analysis version 6.5 (Ingenuity^®^ Systems, www.ingenuity.com). Gene Set Enrichment Analysis (GSEA) [24] was performed against lists of differentially expressed genes for *VHL*-single hit *vs*. *WT, TSC*-single hit *vs*. *WT*, and *VHL*-single hit^VHL knock-down^ *vs*. *VHL*-single hit^pLKO control^ comparisons. Gene sets from MSigDB [24], including positional, curated, motif and computational sets, were tested. Default parameters were chosen, except that maximum intensity of probes was selected while collapsing probe sets for each gene.

To discover a common theme of differential gene expression patterns across previously published transcriptomic profiling studies of RCC subtypes to that of *VHL* one-hit kidney epithelial cells, we obtained raw gene expression data from previous studies (Supplemental Table 1). The renal cancer subtypes evaluated were ccRCC, oncocytoma, chromophobe, papillary, transitional cell and Wilms’ tumor. Data were analyzed as previously outlined; and since the primary focus was on observing up- or down-regulation, no p-value or fold-change cutoffs were enforced.

### Glucose uptake assay

Five WT and five *VHL*-single hit cultures were seeded in 6-well plates at ~70% confluency and incubated with serum-free, low glucose DMEM. 2-NBDG (2-(N-(7-Nitrobenz-2-oxa-1,3-diazol-4-yl)Amino)-2-Deoxyglucose) (Life Technologies) was dissolved in sterile Milli-Q water at a concentration of 100 mM. After 24 h, cells were incubated for 30 min at 37°C with 2-NBDG (final concentration: 100 μM). 2-NBDG uptake reaction was stopped by removing the media and washing cells once with pre-chilled PBS. Cells were collected, centrifuged and resuspended in 500 μl PBS for flow cytometry analysis.

### Lactate production assay

Lactate levels were quantitated from media of 5 WT and 5 *VHL*-single hit cultures, per Sigma's instructions. Cells were seeded on 6-well plates in ACL-4 complete media; after 24 h, cells were cultured in phenol red-free complete RPMI-1640 overnight; media was then removed from the cells and deproteinized with a 10-kDa MWCO spin filter to remove lactate dehydrogenase. The soluble fraction was assayed directly. Cells were trypsinized and counted for normalization of lactate concentration.

The authors would like to dedicate this article to the memory of Alfred G. Knudson, scientist, mentor and friend, who worked on the manuscript up to his final days and passed away shortly before submission. He will be missed greatly by us and by scientists and clinicians in the fields of oncology and cancer genetics all over the world.

## SUPPLEMENTARY MATERIALS FIGURES AND TABLES






