# Supplementary material for: Haploinsufficiency in tumor predisposition syndromes: altered genomic transcription in morphologically normal cells heterozygous for VHL or TSC mutation
Source: Oncotarget. 2016 Sep 22;8(11):17628–42. doi: 10.18632/oncotarget.12192 (PMC5392274; doi:10.18632/oncotarget.12192)
Supplement: Supplementary file 1 [file oncotarget-08-17628-s001.pdf]

Haploinsufficiency in tumor predisposition syndromes: altered genomic transcription in morphologically normal cells heterozygous for *VHL* or *TSC* mutation

Supplementary Material

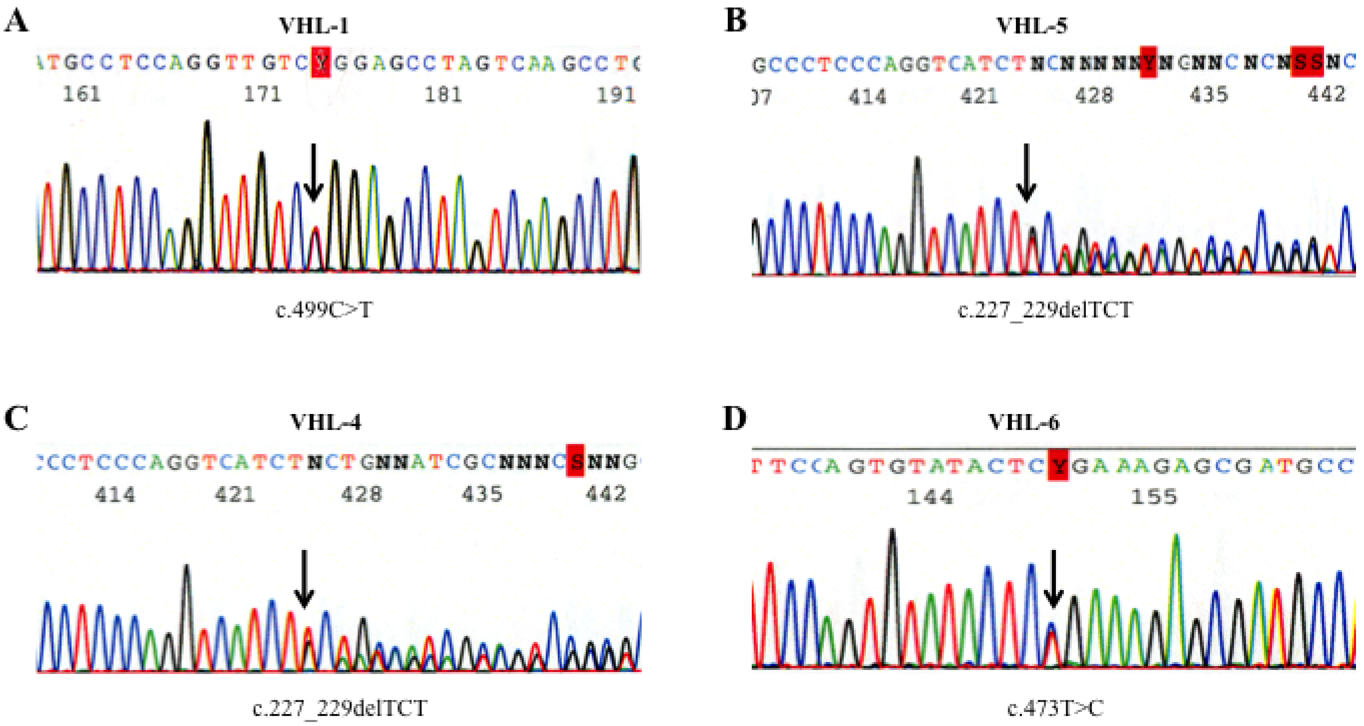

Supplemental Figure 1: Electropherograms showing the VHL sequence variants identified in 5 primary renal epithelial cell cultures from VHL patients.

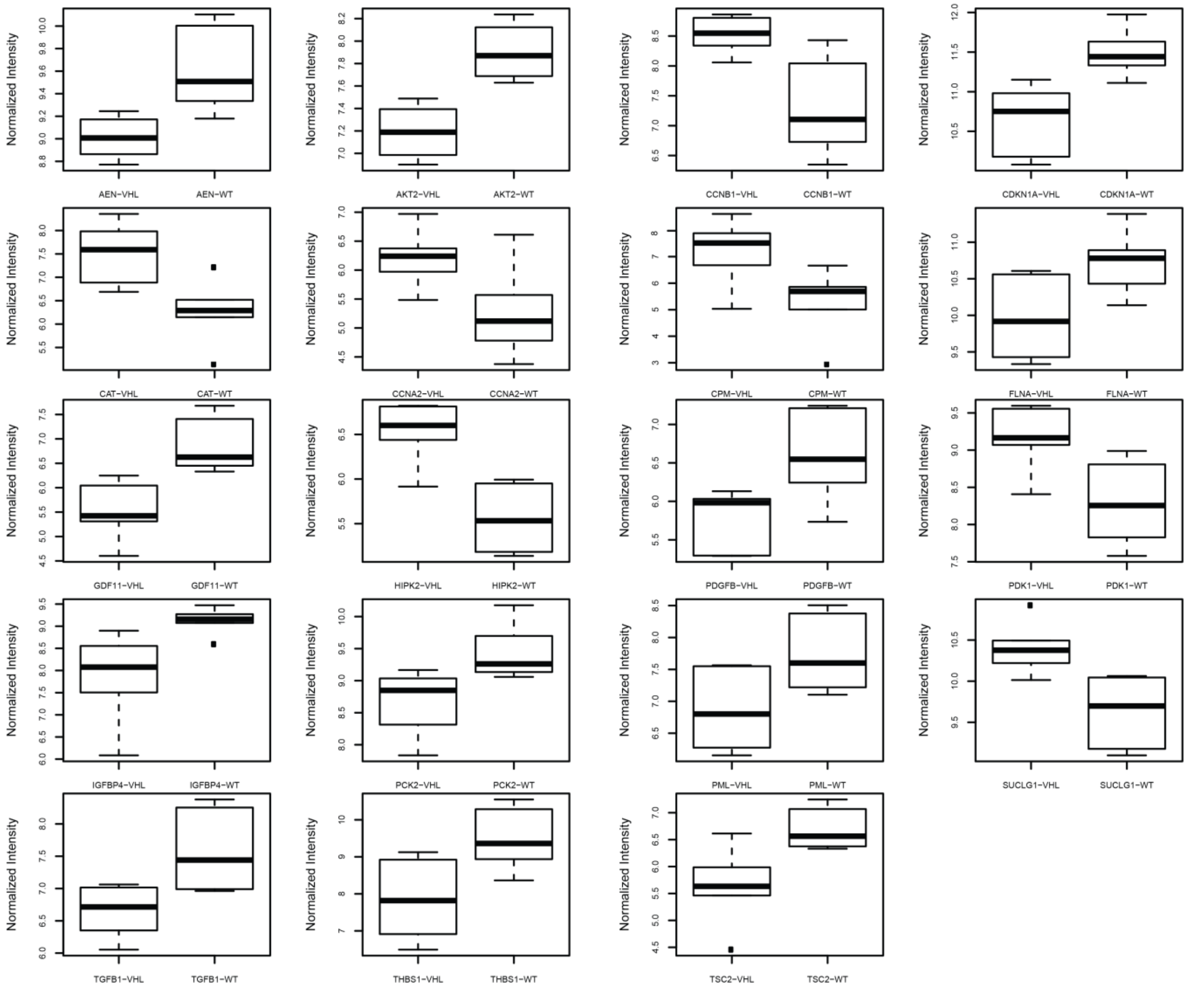

Supplemental Figure 2: Box plots of genes differentially expressed between VHLmut/wt heterozygous cells and WT cells.

[illegible]

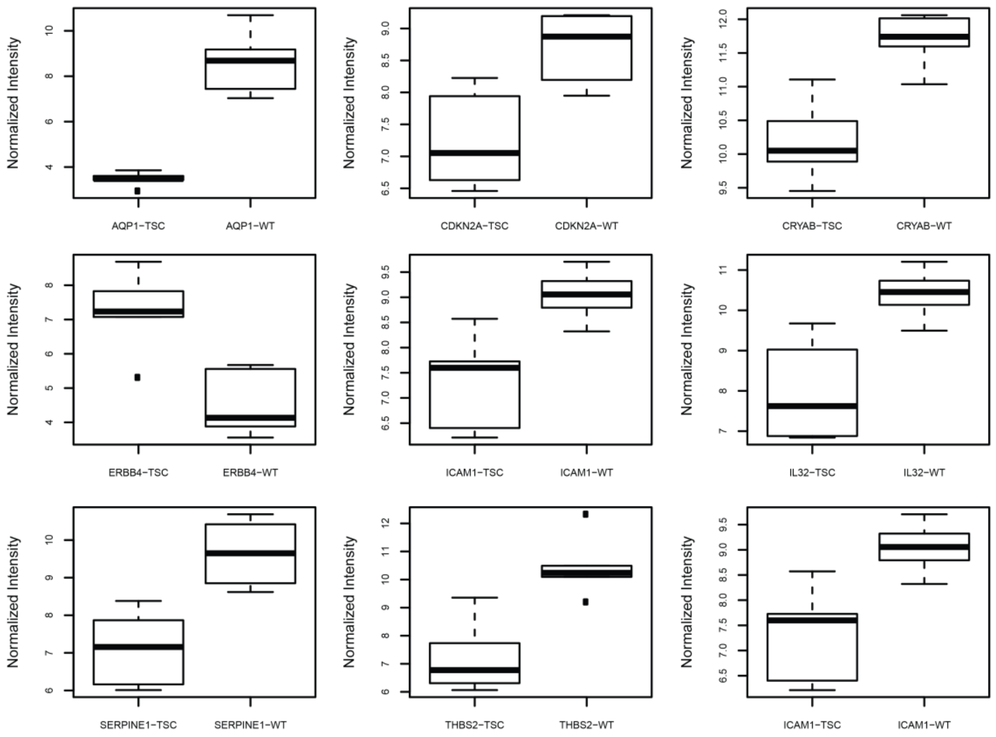

Supplemental Figure 3: Box plots of genes differentially expressed between TSCmut/wt heterozygous cells and WT cells.

Signaling events in Renal cell carcinoma

Citric acid cycle

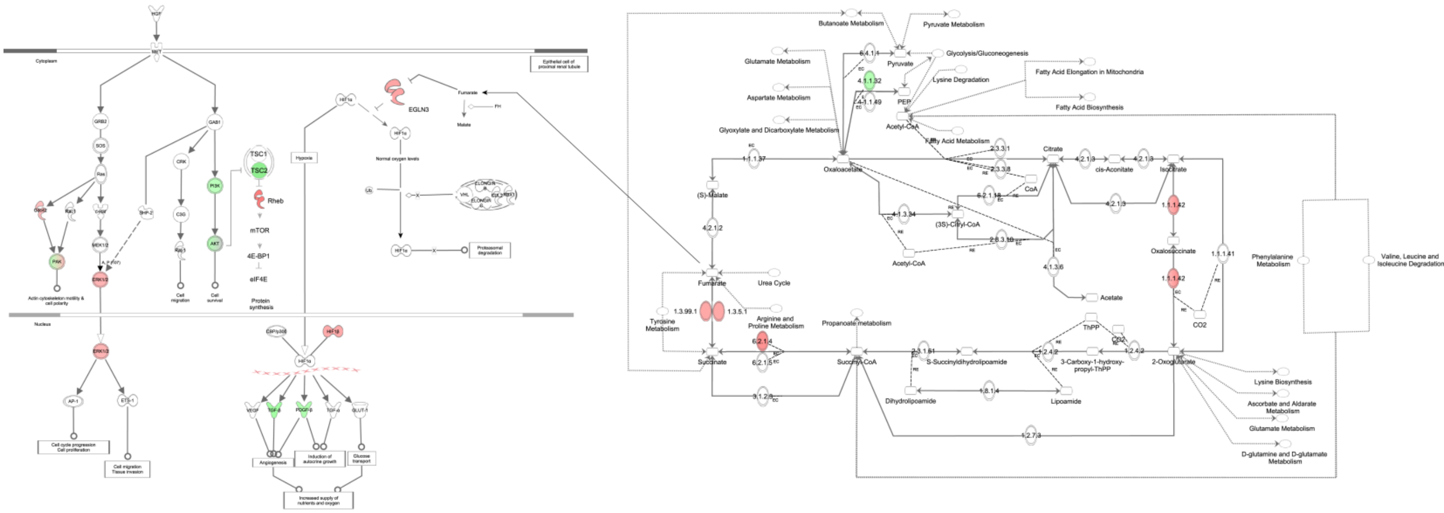

Supplemental Figure 4: Ingenuity Pathway Analysis-based ccRCC pathway showing HIF, AKT/mTOR and TCA cycle pathways.

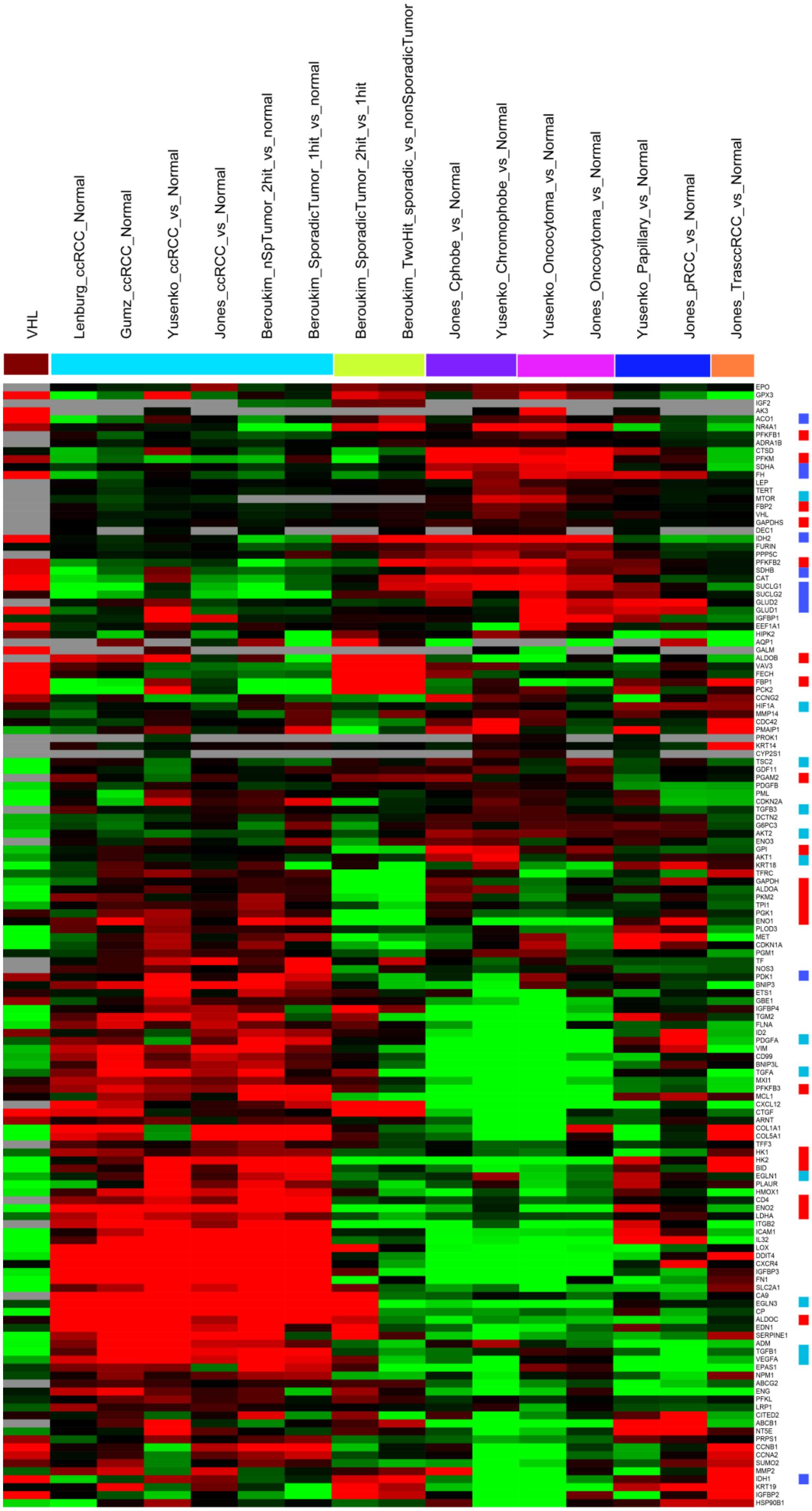

Supplemental Figure 5: Fold-changes of genes from various categories differentially expressed in VHLmut/wt cells or RCC vs. normal kidney epithelium.
