# Supplementary material for: Haploinsufficiency in tumor predisposition syndromes: altered genomic transcription in morphologically normal cells heterozygous for VHL or TSC mutation
Source: Oncotarget. 2016 Sep 22;8(11):17628–42. doi: 10.18632/oncotarget.12192 (PMC5392274; doi:10.18632/oncotarget.12192)
Supplement: Supplementary file 2 [file oncotarget-08-17628-s002.docx]

**Supplemental Table 1:** Gene expression studies on clear cell renal cell carcinoma to which comparisons were made.

| **Study** | **References (PMID)** | **GEO dataset** |
| --- | --- | --- |
| Beroukhim, R., et al., 2009 | 19470766 | GSE14994 |
| Lenburg, M.E., et al., 2003 | 14641932 | GSE781 |
| Gumz, M.L., et al. , 2007 | 17699851 | GSE6344 |
| Yusenko, M.V., et al., 2009 | 19445733 | GSE11151 |
| Jones, J., et al., 2005 | 16115910 | GSE15641 |

**Supplemental Table 2:** *VHL* sequence variants identified in 5 primary renal epithelial cell lines from VHL patients.

| **SAMPLE NAME** | **EXON** | **VARIANT** | **PROTEIN** | **CLASSIFICATION** |
| --- | --- | --- | --- | --- |
| VHL-1 | Exon 3 | c.[499C>T];[=] | p.[Arg167Trp];[=] | Pathogenic^1^ |
| VHL-3 |  | No sequence variants identified |  |  |
| VHL-4 | Exon 1 | c.[227_229delTCT];[=] | p.[Phe76del];[=] | Pathogenic ^1,2,3^ |
| VHL-5 | Exon 1 | c.[21C>A];[=]  c.[227_229delTCT];[=] | p.[Asn7Lys];[=]  p.[Phe76del];[=] | Likely not pathogenic ^4^  Pathogenic ^123^ |
| VHL-6 | Exon 3 | c.[473T>C];[=] | p.[Leu158Pro];[=] | Pathogenic ^2,5^ |

1. Maher et al. 1996;Woodward et al. 1997.
2. Zbar et al. 1996.
3. Dalgliesh et al. 2010.
4. <http://www.1000genomes.org/>
5. Feldman et al. 1999

**Supplemental Table 5 – VHL validation result**

|  | |  |  |  |
| --- | --- | --- | --- | --- |
| **Gene** | **FDR** | **Fold Change** | **Affy Fold change** | **Taq Man assay** |
| AEN | 0.164912281 | -0.937777778 | -0.595173447 | Hs00224322_m1 |
| AKT2 | 0.111904762 | -0.878333332 | -0.712087834 | Hs01086102_m1 |
| CAT | 0.223809524 | 0.3734375 | 1.249123142 | Hs00156308_m1 |
| CCNA2 | 0.164912281 | 0.664145833 | 0.950421219 | Hs00153138_m1 |
| CCNB1 | 0.111904762 | 0.9581875 | 1.233306163 | Hs01030097_m1 |
| CDKN1A | 0.111904762 | -1.1526875 | -0.839864463 | Hs00355782_m1 |
| CDKN2A | 0.164912281 | -1.606062498 | -1.27714483 | Hs00923894_m1 |
| CPM | 0.111904762 | 0.737583334 | 1.895834784 | Hs00266395_m1 |
| FLNA | 0.223809524 | -1.026395833 | -0.774134445 | Hs00924645_m1 |
| GDF11 | 0.164912281 | -0.677395834 | -1.344513435 | Hs00195156_m1 |
| HIPK2 | 0.162770563 | 0.229 | 0.976636582 | Hs00179759_m1 |
| ICAM1 | 0.223809524 | -0.960743056 | -0.885355915 | Hs00164932_m1 |
| IGFBP4 | 0.164912281 | -1.413083333 | -1.255911928 | Hs00181767_m1 |
| IL32 | 0.164912281 | -2.060673612 | -1.407881429 | Hs00170403_m1 |
| PCK2 | 0.111904762 | -0.920652779 | -0.757605481 | Hs00388934_m1 |
| PDGFB | 0.162770563 | -1.464729165 | -0.801973843 | Hs00234042_m1 |
| PDK1 | 0.162770563 | 0.639666666 | 0.8745904 | Hs01561850_m1 |
| PIDD | 0.111904762 | -0.922159723 |  | Hs00388035_m1 |
| PML | 0.164912281 | -1.11529861 | -0.877134461 | Hs00971694_m1 |
| SUCLG1 | 0.223809524 | 0.600361111 | 0.770562132 | Hs00388749_m1 |
| TGFB1 | 0.111904762 | -0.937472221 | -0.925841219 | Hs00998133_m1 |
| THBS1 | 0.223809524 | -1.203638887 | -1.628217978 | Hs00962908_m1 |
| TSC2 | 0.164912281 | -1.0180625 | -1.060024702 | Hs01020387_m1 |
| VAV3 | 0.111904762 | 1.332805556 | 1.085960254 | Hs00196125_m1 |

**Supplemental Table 6 – TSC validation result**

| **Gene** | **FDR** | **Fold Change** | **Affy Fold change** | **Taq Man assay** |
| --- | --- | --- | --- | --- |
| AEN | 0.114774115 | -1.188825 | -0.892689704 | Hs00224322_m1 |
| AKT2 | 0.114774115 | -0.750225 | -0.499380259 | Hs01086102_m1 |
| AQP1 | 0.106575964 | -5.3513375 | -5.155144355 | Hs00166067_m1 |
| CDKN1A | 0.106575964 | -1.3104625 | -1.06268033 | Hs00355782_m1 |
| CDKN2A | 0.106575964 | -1.9144375 | -1.488580598 | Hs00923894_m1 |
| CRYAB | 0.114774115 | -1.97965 | -1.525051766 | Hs00157107_m1 |
| ERBB4 | 0.248677249 | 2.1365125 | 2.736817922 | Hs00955525_m1 |
| FLNA | 0.114774115 | -1.1232375 | -1.157679113 | Hs00924645_m1 |
| GDF11 | 0.248677249 | -0.6872125 | -0.726021881 | Hs00195156_m1 |
| ICAM1 | 0.106575964 | -1.830779167 | -1.689945325 | Hs00164932_m1 |
| IGFBP4 | 0.114774115 | -1.823 | -1.608511313 | Hs00181767_m1 |
| IL32 | 0.106575964 | -2.874695833 | -2.469465546 | Hs00170403_m1 |
| PCK2 | 0.106575964 | -1.048708333 | -0.789023284 | Hs00388934_m1 |
| PDGFB | 0.248677249 | -1.4417375 | -1.103330947 | Hs00234042_m1 |
| PIDD | 0.198941799 | -0.677812501 |  | Hs00388035_m1 |
| PML | 0.114774115 | -1.2634375 | -1.065955209 | Hs00971694_m1 |
| SERPINE1 | 0.248677249 | -1.977033333 | -2.522634847 | Hs01126604_m1 |
| TGFB1 | 0.248677249 | -0.699475 | -0.428533279 | Hs00998133_m1 |
| THBS2 | 0.248677249 | -3.084362501 | -3.262300992 | Hs01568063_m1 |
| TSC2 | 0.106575964 | -1.421604167 | -0.95297149 | Hs01020387_m1 |
| WT1 | 0.198941799 | -2.449025 | -2.349411961 | Hs01103749_m1 |

**Supplemental Table 8.** Table showing Gene ontology enrichment analysis for up and down regulated genes in cells with abrogated one copy VHL in one-hit cells with shRNA. A - shows all enriched categories for up-regulated genes (VHL-KO vs. onehit). B - Table showing all enriched categories for down-regualted genes (VHL-KO vs. onehit VHL cell). Notation: ID: GO biological process ID; *p* – p-value; O – odds ratio; Exp – Expresed count; Obs – Observed count; size – size of the category; GO term – name of GO biological process.

| ID | *p* | O | Exp | Obs | size | GO Term |
| --- | --- | --- | --- | --- | --- | --- |
| GO:0002486 | 0 | Inf | 0 | 3 | 3 | antigen processing and presentation of endogenous peptide antigen via MHC class I via ER pathway, TAP-independent |
| GO:0009597 | 0 | Inf | 0 | 3 | 3 | detection of virus |
| GO:0034343 | 0 | Inf | 0 | 3 | 3 | type III interferon production |
| GO:0034344 | 0 | Inf | 0 | 3 | 3 | regulation of type III interferon production |
| GO:0002480 | 0 | 52.56 | 1 | 7 | 9 | antigen processing and presentation of exogenous peptide antigen via MHC class I, TAP-independent |
| GO:0002483 | 0 | 52.56 | 1 | 7 | 9 | antigen processing and presentation of endogenous peptide antigen |
| GO:0019885 | 0 | 45 | 1 | 6 | 8 | antigen processing and presentation of endogenous peptide antigen via MHC class I |
| GO:0035456 | 0 | 41.481 | 1 | 11 | 15 | response to interferon-beta |
| GO:0019883 | 0 | 40.089 | 1 | 8 | 11 | antigen processing and presentation of endogenous antigen |
| GO:2000109 | 0 | 19.953 | 0 | 4 | 7 | regulation of macrophage apoptotic process |
| GO:0045071 | 0 | 19.035 | 2 | 20 | 36 | negative regulation of viral genome replication |
| GO:0035458 | 0 | 18.726 | 1 | 5 | 9 | cellular response to interferon-beta |
| GO:0071888 | 0 | 18.726 | 1 | 5 | 9 | macrophage apoptotic process |
| GO:0035455 | 0 | 16.925 | 1 | 9 | 17 | response to interferon-alpha |
| GO:0060337 | 0 | 14.298 | 5 | 36 | 75 | type I interferon signaling pathway |
| GO:0071357 | 0 | 14.298 | 5 | 36 | 75 | cellular response to type I interferon |
| GO:0034340 | 0 | 13.939 | 5 | 36 | 76 | response to type I interferon |
| GO:0001916 | 0 | 13.819 | 1 | 11 | 23 | positive regulation of T cell mediated cytotoxicity |
| GO:0032727 | 0 | 12.482 | 1 | 5 | 11 | positive regulation of interferon-alpha production |
| GO:0001914 | 0 | 11.316 | 2 | 12 | 28 | regulation of T cell mediated cytotoxicity |
| GO:0039528 | 0 | 11.245 | 1 | 6 | 14 | cytoplasmic pattern recognition receptor signaling pathway in response to virus |
| GO:0006925 | 0 | 9.995 | 1 | 6 | 15 | inflammatory cell apoptotic process |
| GO:0048525 | 0 | 9.646 | 4 | 24 | 62 | negative regulation of viral process |
| GO:0045069 | 0 | 9.217 | 3 | 20 | 53 | regulation of viral genome replication |
| GO:0032607 | 0 | 8.995 | 1 | 6 | 16 | interferon-alpha production |
| GO:0032647 | 0 | 8.995 | 1 | 6 | 16 | regulation of interferon-alpha production |
| GO:0001913 | 0 | 8.922 | 2 | 13 | 35 | T cell mediated cytotoxicity |
| GO:0002711 | 0 | 8.922 | 2 | 13 | 35 | positive regulation of T cell mediated immunity |
| GO:0098586 | 0 | 8.176 | 1 | 6 | 17 | cellular response to virus |
| GO:0060333 | 0 | 7.964 | 4 | 24 | 70 | interferon-gamma-mediated signaling pathway |
| GO:0032480 | 0 | 7.868 | 2 | 12 | 35 | negative regulation of type I interferon production |
| GO:0034341 | 0 | 7.619 | 7 | 36 | 109 | response to interferon-gamma |
| GO:0071346 | 0 | 7.291 | 6 | 29 | 90 | cellular response to interferon-gamma |
| GO:0001912 | 0 | 7.204 | 2 | 11 | 34 | positive regulation of leukocyte mediated cytotoxicity |
| GO:0002709 | 0 | 7.088 | 3 | 15 | 47 | regulation of T cell mediated immunity |
| GO:0032732 | 0 | 6.563 | 1 | 7 | 23 | positive regulation of interleukin-1 production |
| GO:0019079 | 0 | 6.465 | 4 | 20 | 67 | viral genome replication |
| GO:0002456 | 0 | 6.268 | 4 | 19 | 65 | T cell mediated immunity |
| GO:0001910 | 0 | 6.029 | 3 | 12 | 42 | regulation of leukocyte mediated cytotoxicity |
| GO:0043901 | 0 | 6.018 | 6 | 26 | 92 | negative regulation of multi-organism process |
| GO:0031343 | 0 | 5.915 | 2 | 11 | 39 | positive regulation of cell killing |
| GO:0002753 | 0 | 5.834 | 3 | 12 | 43 | cytoplasmic pattern recognition receptor signaling pathway |
| GO:0002824 | 0 | 5.813 | 3 | 15 | 54 | positive regulation of adaptive immune response based on somatic recombination of immune receptors built from immunoglobulin superfamily domains |
| GO:0051607 | 0 | 5.736 | 12 | 52 | 194 | defense response to virus |
| GO:0002708 | 0 | 5.419 | 3 | 14 | 53 | positive regulation of lymphocyte mediated immunity |
| GO:0002821 | 0 | 5.396 | 4 | 15 | 57 | positive regulation of adaptive immune response |
| GO:0031341 | 0 | 5.165 | 3 | 12 | 47 | regulation of cell killing |
| GO:0002705 | 0 | 5.154 | 3 | 14 | 55 | positive regulation of leukocyte mediated immunity |
| GO:0001909 | 0 | 5.04 | 4 | 16 | 64 | leukocyte mediated cytotoxicity |
| GO:0009615 | 0 | 4.174 | 17 | 57 | 271 | response to virus |
| GO:0032606 | 0 | 4.162 | 7 | 23 | 107 | type I interferon production |
| GO:0098542 | 0 | 4.139 | 20 | 67 | 323 | defense response to other organism |
| GO:0032479 | 0 | 4.025 | 7 | 22 | 105 | regulation of type I interferon production |
| GO:0045088 | 0 | 3.935 | 16 | 52 | 258 | regulation of innate immune response |
| GO:0001906 | 0 | 3.894 | 5 | 17 | 83 | cell killing |
| GO:0019221 | 0 | 3.876 | 22 | 70 | 356 | cytokine-mediated signaling pathway |
| GO:0050688 | 0 | 3.836 | 5 | 15 | 74 | regulation of defense response to virus |
| GO:0002218 | 0 | 3.751 | 11 | 34 | 173 | activation of innate immune response |
| GO:0045089 | 0 | 3.73 | 13 | 39 | 200 | positive regulation of innate immune response |
| GO:0002221 | 0 | 3.711 | 10 | 32 | 164 | pattern recognition receptor signaling pathway |
| GO:0050792 | 0 | 3.7 | 8 | 26 | 133 | regulation of viral process |
| GO:0097202 | 0 | 3.67 | 5 | 17 | 87 | activation of cysteine-type endopeptidase activity |
| GO:0002758 | 0 | 3.655 | 10 | 32 | 166 | innate immune response-activating signal transduction |
| GO:0002460 | 0 | 3.589 | 12 | 36 | 190 | adaptive immune response based on somatic recombination of immune receptors built from immunoglobulin superfamily domains |
| GO:0002831 | 0 | 3.507 | 6 | 19 | 101 | regulation of response to biotic stimulus |
| GO:0002822 | 0 | 3.445 | 6 | 18 | 97 | regulation of adaptive immune response based on somatic recombination of immune receptors built from immunoglobulin superfamily domains |
| GO:0002252 | 0 | 3.412 | 34 | 94 | 538 | immune effector process |
| GO:0071345 | 0 | 3.406 | 29 | 81 | 460 | cellular response to cytokine stimulus |
| GO:0006919 | 0 | 3.376 | 5 | 15 | 82 | activation of cysteine-type endopeptidase activity involved in apoptotic process |
| GO:0045087 | 0 | 3.364 | 48 | 129 | 766 | innate immune response |
| GO:0002706 | 0 | 3.354 | 6 | 16 | 88 | regulation of lymphocyte mediated immunity |
| GO:0002250 | 0 | 3.346 | 14 | 40 | 224 | adaptive immune response |
| GO:0034162 | 0 | 3.317 | 5 | 13 | 72 | toll-like receptor 9 signaling pathway |
| GO:0002819 | 0 | 3.304 | 7 | 19 | 106 | regulation of adaptive immune response |
| GO:0034097 | 0 | 3.289 | 35 | 94 | 554 | response to cytokine |
| GO:0002449 | 0 | 3.201 | 11 | 30 | 173 | lymphocyte mediated immunity |
| GO:0034142 | 0 | 3.2 | 6 | 18 | 103 | toll-like receptor 4 signaling pathway |
| GO:0002224 | 0 | 3.141 | 9 | 24 | 140 | toll-like receptor signaling pathway |
| GO:0043123 | 0 | 3.139 | 9 | 25 | 146 | positive regulation of I-kappaB kinase/NF-kappaB signaling |
| GO:0031349 | 0 | 3.099 | 17 | 44 | 263 | positive regulation of defense response |
| GO:0002703 | 0 | 3.089 | 7 | 19 | 112 | regulation of leukocyte mediated immunity |
| GO:0043903 | 0 | 3.064 | 10 | 26 | 155 | regulation of symbiosis, encompassing mutualism through parasitism |
| GO:0043122 | 0 | 3.06 | 13 | 34 | 204 | regulation of I-kappaB kinase/NF-kappaB signaling |
| GO:0060759 | 0 | 3.055 | 6 | 16 | 95 | regulation of response to cytokine stimulus |
| GO:2001056 | 0 | 3.024 | 7 | 19 | 114 | positive regulation of cysteine-type endopeptidase activity |
| GO:0002443 | 0 | 2.91 | 14 | 35 | 219 | leukocyte mediated immunity |
| GO:0007249 | 0 | 2.901 | 14 | 36 | 226 | I-kappaB kinase/NF-kappaB signaling |
| GO:0043207 | 0 | 2.897 | 38 | 92 | 600 | response to external biotic stimulus |
| GO:0051707 | 0 | 2.897 | 37 | 91 | 593 | response to other organism |
| GO:0043900 | 0 | 2.877 | 16 | 39 | 247 | regulation of multi-organism process |
| GO:0010950 | 0 | 2.871 | 7 | 19 | 119 | positive regulation of endopeptidase activity |
| GO:0031347 | 0 | 2.866 | 30 | 74 | 481 | regulation of defense response |
| GO:0002699 | 0 | 2.861 | 7 | 18 | 113 | positive regulation of immune effector process |
| GO:0031638 | 0 | 2.85 | 7 | 17 | 107 | zymogen activation |
| GO:0009607 | 0 | 2.828 | 39 | 94 | 626 | response to biotic stimulus |
| GO:0006955 | 0 | 2.808 | 78 | 175 | 1240 | immune response |
| GO:0050778 | 0 | 2.79 | 29 | 69 | 457 | positive regulation of immune response |
| GO:0010952 | 0 | 2.76 | 8 | 19 | 123 | positive regulation of peptidase activity |
| GO:0002697 | 0 | 2.697 | 16 | 37 | 247 | regulation of immune effector process |
| GO:0006952 | 0 | 2.612 | 83 | 175 | 1313 | defense response |
| GO:0002684 | 0 | 2.612 | 40 | 90 | 638 | positive regulation of immune system process |
| GO:0002253 | 0 | 2.556 | 24 | 54 | 381 | activation of immune response |
| GO:0050776 | 0 | 2.43 | 45 | 95 | 717 | regulation of immune response |
| GO:0001819 | 0 | 2.416 | 16 | 35 | 256 | positive regulation of cytokine production |
| GO:0002757 | 0 | 2.4 | 21 | 45 | 333 | immune response-activating signal transduction |
| GO:0032103 | 0 | 2.391 | 11 | 24 | 176 | positive regulation of response to external stimulus |
| GO:0002682 | 0 | 2.308 | 66 | 130 | 1046 | regulation of immune system process |
| GO:0050867 | 0 | 2.296 | 15 | 32 | 244 | positive regulation of cell activation |
| GO:2000116 | 0 | 2.294 | 12 | 25 | 190 | regulation of cysteine-type endopeptidase activity |
| GO:0051251 | 0 | 2.259 | 14 | 28 | 216 | positive regulation of lymphocyte activation |
| GO:0001817 | 0 | 2.241 | 28 | 56 | 442 | regulation of cytokine production |
| GO:0002696 | 0 | 2.233 | 15 | 30 | 234 | positive regulation of leukocyte activation |
| GO:0001816 | 0 | 2.214 | 31 | 62 | 496 | cytokine production |
| GO:0002376 | 0 | 2.137 | 126 | 222 | 2010 | immune system process |
| GO:0080134 | 0 | 2.089 | 53 | 99 | 849 | regulation of response to stress |
| GO:0051249 | 0 | 2.072 | 19 | 37 | 309 | regulation of lymphocyte activation |
| GO:0002694 | 0 | 2.07 | 22 | 42 | 352 | regulation of leukocyte activation |
| GO:0002764 | 0 | 2.06 | 28 | 52 | 440 | immune response-regulating signaling pathway |
| GO:0009617 | 0 | 2.059 | 22 | 41 | 345 | response to bacterium |
| GO:0050865 | 0 | 1.989 | 24 | 44 | 382 | regulation of cell activation |
| GO:0032101 | 0 | 1.898 | 33 | 58 | 528 | regulation of response to external stimulus |
| GO:0042110 | 0 | 1.862 | 23 | 40 | 367 | T cell activation |
| GO:0045321 | 0 | 1.858 | 37 | 64 | 595 | leukocyte activation |
| GO:0071310 | 0 | 1.845 | 99 | 161 | 1580 | cellular response to organic substance |
| GO:0048584 | 0 | 1.797 | 86 | 138 | 1368 | positive regulation of response to stimulus |
| GO:0046649 | 0 | 1.795 | 32 | 53 | 505 | lymphocyte activation |
| GO:0051345 | 0 | 1.752 | 30 | 49 | 476 | positive regulation of hydrolase activity |
| GO:1902533 | 0 | 1.739 | 40 | 65 | 640 | positive regulation of intracellular signal transduction |
| GO:0070887 | 0 | 1.73 | 124 | 189 | 1979 | cellular response to chemical stimulus |
| GO:0009605 | 0 | 1.73 | 111 | 170 | 1764 | response to external stimulus |
| GO:0010033 | 0 | 1.662 | 135 | 198 | 2148 | response to organic substance |
| GO:0001775 | 0 | 1.578 | 51 | 75 | 806 | cell activation |
| GO:0006950 | 0 | 1.568 | 193 | 263 | 3066 | response to stress |
| GO:0048583 | 0 | 1.529 | 170 | 231 | 2707 | regulation of response to stimulus |
| GO:0009967 | 0 | 1.518 | 60 | 86 | 959 | positive regulation of signal transduction |
| GO:1902531 | 0 | 1.509 | 79 | 111 | 1256 | regulation of intracellular signal transduction |
| GO:0042221 | 0 | 1.473 | 189 | 248 | 3006 | response to chemical |
| GO:0050896 | 0 | 1.409 | 417 | 488 | 6638 | response to stimulus |
| GO:0007165 | 0 | 1.405 | 272 | 335 | 4325 | signal transduction |
| GO:1901575 | 0 | 1.4 | 127 | 165 | 2021 | organic substance catabolic process |
| GO:0009966 | 0 | 1.391 | 130 | 168 | 2070 | regulation of signal transduction |
| GO:0035556 | 0 | 1.381 | 127 | 163 | 2016 | intracellular signal transduction |
| GO:0009056 | 0 | 1.361 | 142 | 179 | 2250 | catabolic process |
| GO:0023052 | 0 | 1.353 | 301 | 359 | 4784 | signaling |
| GO:0044700 | 0 | 1.353 | 301 | 359 | 4784 | single organism signaling |
| GO:0007154 | 0 | 1.341 | 305 | 362 | 4854 | cell communication |
| GO:0051716 | 0 | 1.318 | 330 | 385 | 5250 | cellular response to stimulus |
| GO:0002484 | 0.001 | 44.849 | 0 | 3 | 4 | antigen processing and presentation of endogenous peptide antigen via MHC class I via ER pathway |
| GO:0034154 | 0.001 | 44.849 | 0 | 3 | 4 | toll-like receptor 7 signaling pathway |
| GO:0070269 | 0.001 | 44.849 | 0 | 3 | 4 | pyroptosis |
| GO:1900246 | 0.001 | 44.849 | 0 | 3 | 4 | positive regulation of RIG-I signaling pathway |
| GO:0046596 | 0.001 | 9.36 | 1 | 5 | 13 | regulation of viral entry into host cell |
| GO:0002833 | 0.001 | 6.423 | 1 | 6 | 20 | positive regulation of response to biotic stimulus |
| GO:0032731 | 0.001 | 5.994 | 1 | 6 | 21 | positive regulation of interleukin-1 beta production |
| GO:0098581 | 0.001 | 5.994 | 1 | 6 | 21 | detection of external biotic stimulus |
| GO:0033028 | 0.001 | 5.249 | 2 | 7 | 27 | myeloid cell apoptotic process |
| GO:0046718 | 0.001 | 5.249 | 2 | 7 | 27 | viral entry into host cell |
| GO:0070534 | 0.001 | 4.998 | 2 | 7 | 28 | protein K63-linked ubiquitination |
| GO:0030890 | 0.001 | 4.618 | 2 | 8 | 34 | positive regulation of B cell proliferation |
| GO:1902476 | 0.001 | 3.975 | 3 | 9 | 43 | chloride transmembrane transport |
| GO:0032612 | 0.001 | 3.861 | 3 | 9 | 44 | interleukin-1 production |
| GO:0006821 | 0.001 | 3.577 | 3 | 10 | 52 | chloride transport |
| GO:0030888 | 0.001 | 3.577 | 3 | 10 | 52 | regulation of B cell proliferation |
| GO:0042787 | 0.001 | 3.223 | 4 | 12 | 68 | protein ubiquitination involved in ubiquitin-dependent protein catabolic process |
| GO:0032481 | 0.001 | 3.156 | 5 | 13 | 75 | positive regulation of type I interferon production |
| GO:0002756 | 0.001 | 3.057 | 5 | 13 | 77 | MyD88-independent toll-like receptor signaling pathway |
| GO:0051701 | 0.001 | 2.838 | 6 | 16 | 101 | interaction with host |
| GO:0001959 | 0.001 | 2.81 | 6 | 14 | 89 | regulation of cytokine-mediated signaling pathway |
| GO:0043280 | 0.001 | 2.65 | 7 | 16 | 107 | positive regulation of cysteine-type endopeptidase activity involved in apoptotic process |
| GO:0051092 | 0.001 | 2.513 | 7 | 17 | 119 | positive regulation of NF-kappaB transcription factor activity |
| GO:0002274 | 0.001 | 2.446 | 8 | 18 | 129 | myeloid leukocyte activation |
| GO:0001818 | 0.001 | 2.389 | 9 | 19 | 139 | negative regulation of cytokine production |
| GO:0002478 | 0.001 | 2.298 | 10 | 21 | 159 | antigen processing and presentation of exogenous peptide antigen |
| GO:0048002 | 0.001 | 2.288 | 11 | 23 | 175 | antigen processing and presentation of peptide antigen |
| GO:0032943 | 0.001 | 2.116 | 13 | 26 | 212 | mononuclear cell proliferation |
| GO:0070661 | 0.001 | 2.027 | 14 | 26 | 220 | leukocyte proliferation |
| GO:0052548 | 0.001 | 1.894 | 18 | 31 | 279 | regulation of endopeptidase activity |
| GO:0002521 | 0.001 | 1.841 | 24 | 41 | 380 | leukocyte differentiation |
| GO:0051223 | 0.001 | 1.834 | 21 | 36 | 334 | regulation of protein transport |
| GO:0044257 | 0.001 | 1.798 | 24 | 41 | 388 | cellular protein catabolic process |
| GO:0030163 | 0.001 | 1.642 | 35 | 54 | 556 | protein catabolic process |
| GO:0023056 | 0.001 | 1.476 | 63 | 88 | 1006 | positive regulation of signaling |
| GO:0051604 | 0.001 | 1.472 | 69 | 95 | 1091 | protein maturation |
| GO:0010647 | 0.001 | 1.469 | 64 | 88 | 1010 | positive regulation of cell communication |
| GO:0016485 | 0.001 | 1.466 | 68 | 94 | 1083 | protein processing |
| GO:0044093 | 0.001 | 1.425 | 73 | 98 | 1158 | positive regulation of molecular function |
| GO:0051704 | 0.001 | 1.376 | 117 | 150 | 1854 | multi-organism process |
| GO:0044248 | 0.001 | 1.348 | 119 | 151 | 1897 | cellular catabolic process |
| GO:0023051 | 0.001 | 1.334 | 145 | 181 | 2313 | regulation of signaling |
| GO:0010646 | 0.001 | 1.318 | 146 | 180 | 2321 | regulation of cell communication |
| GO:0032020 | 0.002 | 22.423 | 0 | 3 | 5 | ISG15-protein conjugation |
| GO:0039530 | 0.002 | 22.423 | 0 | 3 | 5 | MDA-5 signaling pathway |
| GO:0046597 | 0.002 | 11.97 | 1 | 4 | 9 | negative regulation of viral entry into host cell |
| GO:0071360 | 0.002 | 11.97 | 1 | 4 | 9 | cellular response to exogenous dsRNA |
| GO:0006622 | 0.002 | 9.974 | 1 | 4 | 10 | protein targeting to lysosome |
| GO:0055094 | 0.002 | 9.974 | 1 | 4 | 10 | response to lipoprotein particle |
| GO:0071850 | 0.002 | 9.974 | 1 | 4 | 10 | mitotic cell cycle arrest |
| GO:0032728 | 0.002 | 5.619 | 1 | 6 | 22 | positive regulation of interferon-beta production |
| GO:0033032 | 0.002 | 5.619 | 1 | 6 | 22 | regulation of myeloid cell apoptotic process |
| GO:0032652 | 0.002 | 4.139 | 2 | 8 | 37 | regulation of interleukin-1 production |
| GO:0060338 | 0.002 | 4.139 | 2 | 8 | 37 | regulation of type I interferon-mediated signaling pathway |
| GO:0007229 | 0.002 | 2.864 | 5 | 12 | 75 | integrin-mediated signaling pathway |
| GO:0034138 | 0.002 | 2.834 | 5 | 13 | 82 | toll-like receptor 3 signaling pathway |
| GO:0032946 | 0.002 | 2.566 | 6 | 15 | 103 | positive regulation of mononuclear cell proliferation |
| GO:0070665 | 0.002 | 2.509 | 7 | 15 | 105 | positive regulation of leukocyte proliferation |
| GO:0042742 | 0.002 | 2.299 | 9 | 18 | 136 | defense response to bacterium |
| GO:0032944 | 0.002 | 2.203 | 10 | 20 | 157 | regulation of mononuclear cell proliferation |
| GO:0019884 | 0.002 | 2.186 | 10 | 21 | 166 | antigen processing and presentation of exogenous antigen |
| GO:0009306 | 0.002 | 2.119 | 12 | 23 | 187 | protein secretion |
| GO:0043281 | 0.002 | 2.116 | 11 | 22 | 179 | regulation of cysteine-type endopeptidase activity involved in apoptotic process |
| GO:0046651 | 0.002 | 2.043 | 13 | 25 | 210 | lymphocyte proliferation |
| GO:0019882 | 0.002 | 2.021 | 13 | 25 | 212 | antigen processing and presentation |
| GO:0050863 | 0.002 | 1.964 | 15 | 27 | 235 | regulation of T cell activation |
| GO:0030335 | 0.002 | 1.9 | 16 | 29 | 260 | positive regulation of cell migration |
| GO:2000147 | 0.002 | 1.867 | 17 | 29 | 264 | positive regulation of cell motility |
| GO:0052547 | 0.002 | 1.848 | 18 | 31 | 285 | regulation of peptidase activity |
| GO:0070201 | 0.002 | 1.701 | 24 | 39 | 387 | regulation of establishment of protein localization |
| GO:0006954 | 0.002 | 1.628 | 33 | 50 | 518 | inflammatory response |
| GO:0009057 | 0.002 | 1.468 | 58 | 81 | 928 | macromolecule catabolic process |
| GO:0008219 | 0.002 | 1.333 | 112 | 141 | 1783 | cell death |
| GO:0016265 | 0.002 | 1.331 | 112 | 141 | 1785 | death |
| GO:0009595 | 0.003 | 4.994 | 2 | 6 | 24 | detection of biotic stimulus |
| GO:0032459 | 0.003 | 4.994 | 2 | 6 | 24 | regulation of protein oligomerization |
| GO:0030260 | 0.003 | 4.197 | 2 | 7 | 32 | entry into host cell |
| GO:0044409 | 0.003 | 4.197 | 2 | 7 | 32 | entry into host |
| GO:0051806 | 0.003 | 4.197 | 2 | 7 | 32 | entry into cell of other organism involved in symbiotic interaction |
| GO:0051828 | 0.003 | 4.197 | 2 | 7 | 32 | entry into other organism involved in symbiotic interaction |
| GO:0052126 | 0.003 | 4.197 | 2 | 7 | 32 | movement in host environment |
| GO:0052192 | 0.003 | 4.197 | 2 | 7 | 32 | movement in environment of other organism involved in symbiotic interaction |
| GO:0032611 | 0.003 | 3.75 | 3 | 8 | 40 | interleukin-1 beta production |
| GO:0042100 | 0.003 | 2.775 | 5 | 12 | 77 | B cell proliferation |
| GO:0031348 | 0.003 | 2.538 | 6 | 14 | 97 | negative regulation of defense response |
| GO:0070663 | 0.003 | 2.139 | 10 | 20 | 161 | regulation of leukocyte proliferation |
| GO:0030522 | 0.003 | 1.889 | 15 | 26 | 234 | intracellular receptor signaling pathway |
| GO:0051272 | 0.003 | 1.82 | 17 | 29 | 270 | positive regulation of cellular component movement |
| GO:0032386 | 0.003 | 1.727 | 22 | 36 | 352 | regulation of intracellular transport |
| GO:0044265 | 0.003 | 1.48 | 46 | 64 | 724 | cellular macromolecule catabolic process |
| GO:0012501 | 0.003 | 1.332 | 101 | 127 | 1601 | programmed cell death |
| GO:0007166 | 0.003 | 1.278 | 158 | 190 | 2515 | cell surface receptor signaling pathway |
| GO:0002182 | 0.004 | Inf | 0 | 2 | 2 | cytoplasmic translational elongation |
| GO:0033364 | 0.004 | Inf | 0 | 2 | 2 | mast cell secretory granule organization |
| GO:0055099 | 0.004 | Inf | 0 | 2 | 2 | response to high density lipoprotein particle |
| GO:1900245 | 0.004 | Inf | 0 | 2 | 2 | positive regulation of MDA-5 signaling pathway |
| GO:1900247 | 0.004 | Inf | 0 | 2 | 2 | regulation of cytoplasmic translational elongation |
| GO:1900248 | 0.004 | Inf | 0 | 2 | 2 | negative regulation of cytoplasmic translational elongation |
| GO:0002664 | 0.004 | 8.549 | 1 | 4 | 11 | regulation of T cell tolerance induction |
| GO:0006623 | 0.004 | 8.549 | 1 | 4 | 11 | protein targeting to vacuole |
| GO:0019852 | 0.004 | 8.549 | 1 | 4 | 11 | L-ascorbic acid metabolic process |
| GO:0039529 | 0.004 | 8.549 | 1 | 4 | 11 | RIG-I signaling pathway |
| GO:0072666 | 0.004 | 8.549 | 1 | 4 | 11 | establishment of protein localization to vacuole |
| GO:0090399 | 0.004 | 8.549 | 1 | 4 | 11 | replicative senescence |
| GO:0045824 | 0.004 | 5.758 | 1 | 5 | 18 | negative regulation of innate immune response |
| GO:0098543 | 0.004 | 5.758 | 1 | 5 | 18 | detection of other organism |
| GO:0030224 | 0.004 | 4.731 | 2 | 6 | 25 | monocyte differentiation |
| GO:0060330 | 0.004 | 4.731 | 2 | 6 | 25 | regulation of response to interferon-gamma |
| GO:0031497 | 0.004 | 2.478 | 6 | 14 | 99 | chromatin assembly |
| GO:0048520 | 0.004 | 2.449 | 6 | 14 | 100 | positive regulation of behavior |
| GO:0050670 | 0.004 | 2.09 | 10 | 19 | 156 | regulation of lymphocyte proliferation |
| GO:0002683 | 0.004 | 1.95 | 13 | 23 | 201 | negative regulation of immune system process |
| GO:0051091 | 0.004 | 1.939 | 13 | 23 | 202 | positive regulation of sequence-specific DNA binding transcription factor activity |
| GO:0032880 | 0.004 | 1.604 | 28 | 43 | 450 | regulation of protein localization |
| GO:0048585 | 0.004 | 1.417 | 59 | 79 | 932 | negative regulation of response to stimulus |
| GO:0015031 | 0.004 | 1.356 | 81 | 104 | 1283 | protein transport |
| GO:0032879 | 0.004 | 1.326 | 98 | 123 | 1554 | regulation of localization |
| GO:0002517 | 0.005 | 7.479 | 1 | 4 | 12 | T cell tolerance induction |
| GO:0045351 | 0.005 | 7.479 | 1 | 4 | 12 | type I interferon biosynthetic process |
| GO:0090162 | 0.005 | 7.479 | 1 | 4 | 12 | establishment of epithelial cell polarity |
| GO:0043516 | 0.005 | 4.494 | 2 | 6 | 26 | regulation of DNA damage response, signal transduction by p53 class mediator |
| GO:0032651 | 0.005 | 3.886 | 2 | 7 | 34 | regulation of interleukin-1 beta production |
| GO:0071230 | 0.005 | 3.428 | 3 | 8 | 43 | cellular response to amino acid stimulus |
| GO:0072527 | 0.005 | 2.754 | 4 | 11 | 71 | pyrimidine-containing compound metabolic process |
| GO:0050671 | 0.005 | 2.393 | 6 | 14 | 102 | positive regulation of lymphocyte proliferation |
| GO:0050870 | 0.005 | 2.023 | 11 | 20 | 169 | positive regulation of T cell activation |
| GO:0040017 | 0.005 | 1.76 | 17 | 29 | 278 | positive regulation of locomotion |
| GO:0006915 | 0.005 | 1.311 | 99 | 124 | 1582 | apoptotic process |
| GO:0002690 | 0.006 | 3 | 3 | 9 | 54 | positive regulation of leukocyte chemotaxis |
| GO:0051209 | 0.006 | 2.664 | 5 | 11 | 73 | release of sequestered calcium ion into cytosol |
| GO:0051282 | 0.006 | 2.664 | 5 | 11 | 73 | regulation of sequestering of calcium ion |
| GO:0051283 | 0.006 | 2.664 | 5 | 11 | 73 | negative regulation of sequestering of calcium ion |
| GO:0035666 | 0.006 | 2.622 | 5 | 11 | 74 | TRIF-dependent toll-like receptor signaling pathway |
| GO:0002755 | 0.006 | 2.539 | 5 | 12 | 83 | MyD88-dependent toll-like receptor signaling pathway |
| GO:0002474 | 0.006 | 2.412 | 6 | 13 | 94 | antigen processing and presentation of peptide antigen via MHC class I |
| GO:0030334 | 0.006 | 1.56 | 29 | 43 | 461 | regulation of cell migration |
| GO:0040012 | 0.006 | 1.518 | 33 | 48 | 528 | regulation of locomotion |
| GO:0043085 | 0.006 | 1.39 | 60 | 79 | 948 | positive regulation of catalytic activity |
| GO:0051049 | 0.006 | 1.355 | 71 | 92 | 1132 | regulation of transport |
| GO:0032610 | 0.007 | 11.21 | 0 | 3 | 7 | interleukin-1 alpha production |
| GO:0002643 | 0.007 | 6.648 | 1 | 4 | 13 | regulation of tolerance induction |
| GO:0010745 | 0.007 | 6.648 | 1 | 4 | 13 | negative regulation of macrophage derived foam cell differentiation |
| GO:0032462 | 0.007 | 6.648 | 1 | 4 | 13 | regulation of protein homooligomerization |
| GO:0034123 | 0.007 | 6.648 | 1 | 4 | 13 | positive regulation of toll-like receptor signaling pathway |
| GO:0043517 | 0.007 | 6.648 | 1 | 4 | 13 | positive regulation of DNA damage response, signal transduction by p53 class mediator |
| GO:0072665 | 0.007 | 6.648 | 1 | 4 | 13 | protein localization to vacuole |
| GO:0033198 | 0.007 | 4.989 | 1 | 5 | 20 | response to ATP |
| GO:0034308 | 0.007 | 4.989 | 1 | 5 | 20 | primary alcohol metabolic process |
| GO:0034134 | 0.007 | 2.581 | 5 | 11 | 75 | toll-like receptor 2 signaling pathway |
| GO:0042590 | 0.007 | 2.581 | 5 | 11 | 75 | antigen processing and presentation of exogenous peptide antigen via MHC class I |
| GO:0006334 | 0.007 | 2.469 | 5 | 12 | 85 | nucleosome assembly |
| GO:0071248 | 0.007 | 2.469 | 5 | 12 | 85 | cellular response to metal ion |
| GO:0050921 | 0.007 | 2.436 | 5 | 12 | 86 | positive regulation of chemotaxis |
| GO:0051222 | 0.007 | 1.885 | 12 | 22 | 198 | positive regulation of protein transport |
| GO:0044764 | 0.007 | 1.452 | 41 | 57 | 654 | multi-organism cellular process |
| GO:0044403 | 0.007 | 1.44 | 44 | 61 | 706 | symbiosis, encompassing mutualism through parasitism |
| GO:0044419 | 0.007 | 1.44 | 44 | 61 | 706 | interspecies interaction between organisms |
| GO:0051336 | 0.007 | 1.388 | 57 | 75 | 900 | regulation of hydrolase activity |
| GO:0045184 | 0.007 | 1.315 | 85 | 107 | 1356 | establishment of protein localization |
| GO:0050871 | 0.008 | 2.872 | 4 | 9 | 56 | positive regulation of B cell activation |
| GO:0000041 | 0.008 | 2.403 | 5 | 12 | 87 | transition metal ion transport |
| GO:1902105 | 0.008 | 1.884 | 12 | 21 | 189 | regulation of leukocyte differentiation |
| GO:0051240 | 0.008 | 1.489 | 35 | 50 | 560 | positive regulation of multicellular organismal process |
| GO:0016032 | 0.008 | 1.448 | 41 | 56 | 644 | viral process |
| GO:0006607 | 0.009 | 5.983 | 1 | 4 | 14 | NLS-bearing protein import into nucleus |
| GO:0006825 | 0.009 | 5.983 | 1 | 4 | 14 | copper ion transport |
| GO:0010818 | 0.009 | 5.983 | 1 | 4 | 14 | T cell chemotaxis |
| GO:1901798 | 0.009 | 5.983 | 1 | 4 | 14 | positive regulation of signal transduction by p53 class mediator |
| GO:0001961 | 0.009 | 4.677 | 1 | 5 | 21 | positive regulation of cytokine-mediated signaling pathway |
| GO:0002720 | 0.009 | 4.677 | 1 | 5 | 21 | positive regulation of cytokine production involved in immune response |
| GO:0045022 | 0.009 | 4.677 | 1 | 5 | 21 | early endosome to late endosome transport |
| GO:0035872 | 0.009 | 2.998 | 3 | 8 | 48 | nucleotide-binding domain, leucine rich repeat containing receptor signaling pathway |
| GO:0043331 | 0.009 | 2.812 | 4 | 9 | 57 | response to dsRNA |
| GO:0032388 | 0.009 | 1.933 | 11 | 19 | 167 | positive regulation of intracellular transport |
| GO:0043632 | 0.009 | 1.563 | 26 | 38 | 406 | modification-dependent macromolecule catabolic process |
| GO:2000145 | 0.009 | 1.512 | 31 | 44 | 485 | regulation of cell motility |
| GO:0051050 | 0.009 | 1.484 | 35 | 49 | 550 | positive regulation of transport |
| GO:0070647 | 0.009 | 1.439 | 41 | 57 | 659 | protein modification by small protein conjugation or removal |
| GO:0071702 | 0.009 | 1.254 | 126 | 151 | 2010 | organic substance transport |
| GO:0010742 | 0.01 | 3.744 | 2 | 6 | 30 | macrophage derived foam cell differentiation |
| GO:0072529 | 0.01 | 3.744 | 2 | 6 | 30 | pyrimidine-containing compound catabolic process |
| GO:0090077 | 0.01 | 3.744 | 2 | 6 | 30 | foam cell differentiation |
| GO:0050864 | 0.01 | 2.34 | 6 | 12 | 89 | regulation of B cell activation |
| GO:0071453 | 0.01 | 2.245 | 6 | 13 | 100 | cellular response to oxygen levels |
| GO:0071216 | 0.01 | 2.108 | 8 | 15 | 122 | cellular response to biotic stimulus |
| GO:0042113 | 0.01 | 1.839 | 12 | 21 | 193 | B cell activation |
| GO:0040011 | 0.01 | 1.304 | 83 | 104 | 1326 | locomotion |

**Suppl. Table 8 - B - Table showing all enriched categories for down-regualted genes (VHL-KO vs. onehit VHL cell).**

| **ID** | ***p*** | **O** | **Exp** | **Obs** | **Size** | **GO Term** |
| --- | --- | --- | --- | --- | --- | --- |
| GO:0009157 | 0 | 36.529 | 0 | 4 | 6 | deoxyribonucleoside monophosphate biosynthetic process |
| GO:0060052 | 0 | 18.262 | 0 | 4 | 8 | neurofilament cytoskeleton organization |
| GO:0007076 | 0 | 11.426 | 1 | 5 | 13 | mitotic chromosome condensation |
| GO:0031055 | 0 | 9.785 | 1 | 8 | 23 | chromatin remodeling at centromere |
| GO:0030261 | 0 | 9.196 | 2 | 10 | 30 | chromosome condensation |
| GO:0000083 | 0 | 9.173 | 1 | 8 | 24 | regulation of transcription involved in G1/S transition of mitotic cell cycle |
| GO:0006336 | 0 | 9.162 | 1 | 7 | 21 | DNA replication-independent nucleosome assembly |
| GO:0034080 | 0 | 9.162 | 1 | 7 | 21 | CENP-A containing nucleosome assembly at centromere |
| GO:0034724 | 0 | 9.162 | 1 | 7 | 21 | DNA replication-independent nucleosome organization |
| GO:0008608 | 0 | 8.446 | 1 | 6 | 19 | attachment of spindle microtubules to kinetochore |
| GO:0034508 | 0 | 6.885 | 2 | 9 | 33 | centromere complex assembly |
| GO:0045103 | 0 | 6.668 | 2 | 8 | 30 | intermediate filament-based process |
| GO:0043486 | 0 | 6.105 | 1 | 7 | 28 | histone exchange |
| GO:0051983 | 0 | 6.105 | 1 | 7 | 28 | regulation of chromosome segregation |
| GO:0043044 | 0 | 5.431 | 2 | 8 | 35 | ATP-dependent chromatin remodeling |
| GO:0000819 | 0 | 4.416 | 3 | 12 | 62 | sister chromatid segregation |
| GO:0000070 | 0 | 4.079 | 3 | 10 | 55 | mitotic sister chromatid segregation |
| GO:2000648 | 0 | 3.963 | 3 | 11 | 62 | positive regulation of stem cell proliferation |
| GO:0007059 | 0 | 3.639 | 8 | 25 | 153 | chromosome segregation |
| GO:0072091 | 0 | 3.625 | 4 | 13 | 79 | regulation of stem cell proliferation |
| GO:0002576 | 0 | 3.369 | 4 | 13 | 84 | platelet degranulation |
| GO:0006323 | 0 | 3.307 | 7 | 20 | 132 | DNA packaging |
| GO:0007088 | 0 | 3.28 | 6 | 16 | 106 | regulation of mitosis |
| GO:0051262 | 0 | 3.276 | 4 | 13 | 86 | protein tetramerization |
| GO:0007067 | 0 | 3.188 | 18 | 50 | 349 | mitosis |
| GO:0060560 | 0 | 3.055 | 7 | 19 | 134 | developmental growth involved in morphogenesis |
| GO:0051783 | 0 | 2.825 | 7 | 17 | 128 | regulation of nuclear division |
| GO:0000280 | 0 | 2.795 | 25 | 61 | 480 | nuclear division |
| GO:0048589 | 0 | 2.711 | 15 | 36 | 285 | developmental growth |
| GO:0061448 | 0 | 2.669 | 11 | 26 | 207 | connective tissue development |
| GO:0048285 | 0 | 2.667 | 27 | 62 | 508 | organelle fission |
| GO:0090287 | 0 | 2.658 | 8 | 19 | 151 | regulation of cellular response to growth factor stimulus |
| GO:0051216 | 0 | 2.643 | 9 | 21 | 168 | cartilage development |
| GO:0071103 | 0 | 2.529 | 10 | 22 | 183 | DNA conformation change |
| GO:0007179 | 0 | 2.505 | 9 | 21 | 176 | transforming growth factor beta receptor signaling pathway |
| GO:0071560 | 0 | 2.485 | 11 | 24 | 203 | cellular response to transforming growth factor beta stimulus |
| GO:0051302 | 0 | 2.479 | 11 | 25 | 212 | regulation of cell division |
| GO:0071559 | 0 | 2.471 | 11 | 24 | 204 | response to transforming growth factor beta |
| GO:0000278 | 0 | 2.439 | 44 | 93 | 842 | mitotic cell cycle |
| GO:0007568 | 0 | 2.377 | 11 | 24 | 211 | aging |
| GO:0000226 | 0 | 2.278 | 16 | 33 | 303 | microtubule cytoskeleton organization |
| GO:0007017 | 0 | 2.261 | 24 | 49 | 458 | microtubule-based process |
| GO:0001501 | 0 | 2.256 | 21 | 43 | 401 | skeletal system development |
| GO:0051301 | 0 | 2.227 | 36 | 72 | 693 | cell division |
| GO:0007178 | 0 | 2.216 | 14 | 29 | 272 | transmembrane receptor protein serine/threonine kinase signaling pathway |
| GO:0044763 | 0 | 2.078 | 532 | 616 | 10195 | single-organism cellular process |
| GO:0044699 | 0 | 2.071 | 590 | 653 | 11312 | single-organism process |
| GO:0044770 | 0 | 2.028 | 23 | 43 | 440 | cell cycle phase transition |
| GO:0030198 | 0 | 2.017 | 19 | 35 | 358 | extracellular matrix organization |
| GO:0043062 | 0 | 2.011 | 19 | 35 | 359 | extracellular structure organization |
| GO:0022402 | 0 | 2.008 | 58 | 103 | 1106 | cell cycle process |
| GO:0044772 | 0 | 2.004 | 23 | 42 | 434 | mitotic cell cycle phase transition |
| GO:0010564 | 0 | 1.903 | 22 | 39 | 421 | regulation of cell cycle process |
| GO:0007049 | 0 | 1.817 | 76 | 124 | 1466 | cell cycle |
| GO:0007155 | 0 | 1.724 | 50 | 79 | 951 | cell adhesion |
| GO:0000904 | 0 | 1.719 | 38 | 61 | 729 | cell morphogenesis involved in differentiation |
| GO:0022610 | 0 | 1.716 | 50 | 79 | 955 | biological adhesion |
| GO:0007010 | 0 | 1.714 | 44 | 70 | 843 | cytoskeleton organization |
| GO:0007167 | 0 | 1.689 | 48 | 75 | 917 | enzyme linked receptor protein signaling pathway |
| GO:0071495 | 0 | 1.66 | 44 | 68 | 841 | cellular response to endogenous stimulus |
| GO:0040007 | 0 | 1.64 | 42 | 64 | 798 | growth |
| GO:0009967 | 0 | 1.578 | 50 | 74 | 959 | positive regulation of signal transduction |
| GO:1902589 | 0 | 1.562 | 85 | 122 | 1632 | single-organism organelle organization |
| GO:0006928 | 0 | 1.527 | 77 | 109 | 1476 | cellular component movement |
| GO:0048731 | 0 | 1.477 | 177 | 231 | 3395 | system development |
| GO:0048513 | 0 | 1.459 | 125 | 166 | 2392 | organ development |
| GO:0016043 | 0 | 1.454 | 237 | 297 | 4548 | cellular component organization |
| GO:0007275 | 0 | 1.427 | 214 | 268 | 4101 | multicellular organismal development |
| GO:0071840 | 0 | 1.425 | 242 | 299 | 4642 | cellular component organization or biogenesis |
| GO:0044707 | 0 | 1.418 | 278 | 337 | 5334 | single-multicellular organism process |
| GO:0048856 | 0 | 1.415 | 208 | 260 | 3987 | anatomical structure development |
| GO:0032501 | 0 | 1.409 | 288 | 346 | 5519 | multicellular organismal process |
| GO:0044767 | 0 | 1.399 | 241 | 295 | 4624 | single-organism developmental process |
| GO:0032502 | 0 | 1.392 | 244 | 297 | 4675 | developmental process |
| GO:0031536 | 0.001 | 54.722 | 0 | 3 | 4 | positive regulation of exit from mitosis |
| GO:0009162 | 0.001 | 14.608 | 0 | 4 | 9 | deoxyribonucleoside monophosphate metabolic process |
| GO:0045109 | 0.001 | 9.139 | 1 | 5 | 15 | intermediate filament organization |
| GO:0045104 | 0.001 | 5.827 | 2 | 7 | 29 | intermediate filament cytoskeleton organization |
| GO:0060444 | 0.001 | 5.776 | 1 | 6 | 25 | branching involved in mammary gland duct morphogenesis |
| GO:0014003 | 0.001 | 4.929 | 2 | 7 | 33 | oligodendrocyte development |
| GO:0010464 | 0.001 | 4.442 | 2 | 8 | 41 | regulation of mesenchymal cell proliferation |
| GO:0031577 | 0.001 | 4.232 | 3 | 9 | 48 | spindle checkpoint |
| GO:0009112 | 0.001 | 3.461 | 3 | 10 | 63 | nucleobase metabolic process |
| GO:0045598 | 0.001 | 3.257 | 4 | 11 | 73 | regulation of fat cell differentiation |
| GO:0002062 | 0.001 | 3.196 | 4 | 12 | 81 | chondrocyte differentiation |
| GO:0045995 | 0.001 | 3.188 | 5 | 13 | 88 | regulation of embryonic development |
| GO:0090288 | 0.001 | 3.065 | 5 | 13 | 91 | negative regulation of cellular response to growth factor stimulus |
| GO:0048565 | 0.001 | 2.789 | 6 | 15 | 114 | digestive tract development |
| GO:0055123 | 0.001 | 2.781 | 6 | 16 | 122 | digestive system development |
| GO:0072089 | 0.001 | 2.781 | 6 | 16 | 122 | stem cell proliferation |
| GO:0050679 | 0.001 | 2.679 | 7 | 17 | 134 | positive regulation of epithelial cell proliferation |
| GO:0045444 | 0.001 | 2.466 | 8 | 17 | 144 | fat cell differentiation |
| GO:0007018 | 0.001 | 2.434 | 9 | 19 | 163 | microtubule-based movement |
| GO:0050678 | 0.001 | 2.144 | 12 | 24 | 231 | regulation of epithelial cell proliferation |
| GO:0072001 | 0.001 | 2.112 | 13 | 25 | 244 | renal system development |
| GO:0001655 | 0.001 | 2.076 | 15 | 28 | 278 | urogenital system development |
| GO:0050673 | 0.001 | 2.007 | 14 | 27 | 276 | epithelial cell proliferation |
| GO:0001525 | 0.001 | 1.862 | 19 | 34 | 373 | angiogenesis |
| GO:0030334 | 0.001 | 1.819 | 24 | 41 | 461 | regulation of cell migration |
| GO:0006935 | 0.001 | 1.693 | 31 | 50 | 602 | chemotaxis |
| GO:0042330 | 0.001 | 1.693 | 31 | 50 | 602 | taxis |
| GO:0070848 | 0.001 | 1.642 | 33 | 51 | 631 | response to growth factor |
| GO:0050878 | 0.001 | 1.641 | 32 | 49 | 606 | regulation of body fluid levels |
| GO:0008284 | 0.001 | 1.623 | 36 | 55 | 689 | positive regulation of cell proliferation |
| GO:0006259 | 0.001 | 1.59 | 46 | 68 | 873 | DNA metabolic process |
| GO:0009987 | 0.001 | 1.532 | 652 | 676 | 12492 | cellular process |
| GO:0009719 | 0.001 | 1.476 | 63 | 88 | 1215 | response to endogenous stimulus |
| GO:0008283 | 0.001 | 1.434 | 84 | 113 | 1615 | cell proliferation |
| GO:0009653 | 0.001 | 1.351 | 111 | 140 | 2121 | anatomical structure morphogenesis |
| GO:0051179 | 0.001 | 1.302 | 231 | 272 | 4426 | localization |
| GO:0051290 | 0.002 | 10.433 | 1 | 4 | 11 | protein heterotetramerization |
| GO:0060045 | 0.002 | 6.526 | 1 | 5 | 19 | positive regulation of cardiac muscle cell proliferation |
| GO:0034453 | 0.002 | 4.418 | 2 | 7 | 36 | microtubule anchoring |
| GO:0030071 | 0.002 | 3.856 | 2 | 8 | 46 | regulation of mitotic metaphase/anaphase transition |
| GO:0051289 | 0.002 | 3.75 | 3 | 9 | 53 | protein homotetramerization |
| GO:0060485 | 0.002 | 2.475 | 7 | 16 | 135 | mesenchyme development |
| GO:2000145 | 0.002 | 1.718 | 25 | 41 | 485 | regulation of cell motility |
| GO:0001944 | 0.002 | 1.677 | 28 | 45 | 545 | vasculature development |
| GO:0071363 | 0.002 | 1.617 | 32 | 49 | 614 | cellular response to growth factor stimulus |
| GO:0051094 | 0.002 | 1.566 | 40 | 59 | 764 | positive regulation of developmental process |
| GO:0023056 | 0.002 | 1.492 | 52 | 74 | 1006 | positive regulation of signaling |
| GO:0010647 | 0.002 | 1.486 | 53 | 74 | 1010 | positive regulation of cell communication |
| GO:0032879 | 0.002 | 1.386 | 81 | 106 | 1554 | regulation of localization |
| GO:0009177 | 0.003 | Inf | 0 | 2 | 2 | pyrimidine deoxyribonucleoside monophosphate biosynthetic process |
| GO:0014856 | 0.003 | Inf | 0 | 2 | 2 | skeletal muscle cell proliferation |
| GO:0018277 | 0.003 | Inf | 0 | 2 | 2 | protein deamination |
| GO:0033693 | 0.003 | Inf | 0 | 2 | 2 | neurofilament bundle assembly |
| GO:0044416 | 0.003 | Inf | 0 | 2 | 2 | induction by symbiont of host defense response |
| GO:0046730 | 0.003 | Inf | 0 | 2 | 2 | induction of host immune response by virus |
| GO:0046732 | 0.003 | Inf | 0 | 2 | 2 | active induction of host immune response by virus |
| GO:0060599 | 0.003 | Inf | 0 | 2 | 2 | lateral sprouting involved in mammary gland duct morphogenesis |
| GO:0075528 | 0.003 | Inf | 0 | 2 | 2 | modulation by virus of host immune response |
| GO:0045110 | 0.003 | 18.238 | 0 | 3 | 6 | intermediate filament bundle assembly |
| GO:0050951 | 0.003 | 9.128 | 1 | 4 | 12 | sensory perception of temperature stimulus |
| GO:0032757 | 0.003 | 6.091 | 1 | 5 | 20 | positive regulation of interleukin-8 production |
| GO:0002053 | 0.003 | 4.27 | 2 | 7 | 37 | positive regulation of mesenchymal cell proliferation |
| GO:0007052 | 0.003 | 4.27 | 2 | 7 | 37 | mitotic spindle organization |
| GO:1902099 | 0.003 | 3.757 | 2 | 8 | 47 | regulation of metaphase/anaphase transition of cell cycle |
| GO:0017015 | 0.003 | 2.754 | 5 | 12 | 92 | regulation of transforming growth factor beta receptor signaling pathway |
| GO:0001822 | 0.003 | 2.049 | 11 | 21 | 210 | kidney development |
| GO:0048514 | 0.003 | 1.788 | 18 | 31 | 352 | blood vessel morphogenesis |
| GO:0016477 | 0.003 | 1.472 | 49 | 68 | 933 | cell migration |
| GO:0040011 | 0.003 | 1.406 | 69 | 92 | 1326 | locomotion |
| GO:0051239 | 0.003 | 1.331 | 99 | 125 | 1907 | regulation of multicellular organismal process |
| GO:0060685 | 0.004 | 13.677 | 0 | 3 | 7 | regulation of prostatic bud formation |
| GO:0070633 | 0.004 | 8.113 | 1 | 4 | 13 | transepithelial transport |
| GO:0010463 | 0.004 | 3.573 | 3 | 8 | 49 | mesenchymal cell proliferation |
| GO:0007200 | 0.004 | 3.171 | 3 | 9 | 61 | phospholipase C-activating G-protein coupled receptor signaling pathway |
| GO:0001837 | 0.004 | 2.803 | 4 | 11 | 83 | epithelial to mesenchymal transition |
| GO:0050680 | 0.004 | 2.654 | 5 | 12 | 95 | negative regulation of epithelial cell proliferation |
| GO:0001935 | 0.004 | 2.622 | 5 | 12 | 96 | endothelial cell proliferation |
| GO:0022617 | 0.004 | 2.45 | 6 | 14 | 119 | extracellular matrix disassembly |
| GO:0090092 | 0.004 | 2.203 | 8 | 17 | 159 | regulation of transmembrane receptor protein serine/threonine kinase signaling pathway |
| GO:0043410 | 0.004 | 1.787 | 17 | 29 | 329 | positive regulation of MAPK cascade |
| GO:0007346 | 0.004 | 1.773 | 18 | 30 | 343 | regulation of mitotic cell cycle |
| GO:0006820 | 0.004 | 1.716 | 20 | 33 | 389 | anion transport |
| GO:0007596 | 0.004 | 1.639 | 26 | 40 | 493 | blood coagulation |
| GO:0050817 | 0.004 | 1.628 | 26 | 40 | 496 | coagulation |
| GO:0007599 | 0.004 | 1.621 | 26 | 40 | 498 | hemostasis |
| GO:0042060 | 0.004 | 1.556 | 32 | 48 | 622 | wound healing |
| GO:0072358 | 0.004 | 1.53 | 37 | 54 | 712 | cardiovascular system development |
| GO:0072359 | 0.004 | 1.53 | 37 | 54 | 712 | circulatory system development |
| GO:0051128 | 0.004 | 1.369 | 76 | 99 | 1463 | regulation of cellular component organization |
| GO:0006996 | 0.004 | 1.289 | 129 | 156 | 2466 | organelle organization |
| GO:0006206 | 0.005 | 4.388 | 2 | 6 | 31 | pyrimidine nucleobase metabolic process |
| GO:0007091 | 0.005 | 3.767 | 2 | 7 | 41 | metaphase/anaphase transition of mitotic cell cycle |
| GO:0014855 | 0.005 | 3.767 | 2 | 7 | 41 | striated muscle cell proliferation |
| GO:0010634 | 0.005 | 2.864 | 4 | 10 | 74 | positive regulation of epithelial cell migration |
| GO:0007051 | 0.005 | 2.654 | 5 | 11 | 87 | spindle organization |
| GO:0051896 | 0.005 | 2.561 | 5 | 12 | 98 | regulation of protein kinase B signaling |
| GO:0014031 | 0.005 | 2.531 | 5 | 12 | 99 | mesenchymal cell development |
| GO:0048762 | 0.005 | 2.435 | 6 | 13 | 111 | mesenchymal cell differentiation |
| GO:0060828 | 0.005 | 2.278 | 7 | 15 | 136 | regulation of canonical Wnt signaling pathway |
| GO:0071774 | 0.005 | 2.013 | 11 | 20 | 203 | response to fibroblast growth factor |
| GO:0030335 | 0.005 | 1.876 | 14 | 24 | 260 | positive regulation of cell migration |
| GO:0022411 | 0.005 | 1.679 | 21 | 34 | 409 | cellular component disassembly |
| GO:0051270 | 0.005 | 1.588 | 28 | 43 | 546 | regulation of cellular component movement |
| GO:0071822 | 0.005 | 1.379 | 66 | 86 | 1257 | protein complex subunit organization |
| GO:0009888 | 0.005 | 1.367 | 70 | 91 | 1342 | tissue development |
| GO:0030154 | 0.005 | 1.264 | 149 | 177 | 2856 | cell differentiation |
| GO:0001556 | 0.006 | 6.637 | 1 | 4 | 15 | oocyte maturation |
| GO:0007567 | 0.006 | 6.637 | 1 | 4 | 15 | parturition |
| GO:0048710 | 0.006 | 5.074 | 1 | 5 | 23 | regulation of astrocyte differentiation |
| GO:0051303 | 0.006 | 5.074 | 1 | 5 | 23 | establishment of chromosome localization |
| GO:0044784 | 0.006 | 3.659 | 2 | 7 | 42 | metaphase/anaphase transition of cell cycle |
| GO:0010770 | 0.006 | 3.557 | 2 | 7 | 43 | positive regulation of cell morphogenesis involved in differentiation |
| GO:0045778 | 0.006 | 3.557 | 2 | 7 | 43 | positive regulation of ossification |
| GO:0060688 | 0.006 | 3.254 | 3 | 8 | 53 | regulation of morphogenesis of a branching structure |
| GO:0002688 | 0.006 | 2.997 | 3 | 9 | 64 | regulation of leukocyte chemotaxis |
| GO:0000086 | 0.006 | 2.113 | 9 | 17 | 165 | G2/M transition of mitotic cell cycle |
| GO:0044839 | 0.006 | 2.113 | 9 | 17 | 165 | cell cycle G2/M phase transition |
| GO:0030323 | 0.006 | 2.098 | 9 | 17 | 166 | respiratory tube development |
| GO:1901988 | 0.006 | 2.044 | 9 | 18 | 180 | negative regulation of cell cycle phase transition |
| GO:2000147 | 0.006 | 1.844 | 14 | 24 | 264 | positive regulation of cell motility |
| GO:0051130 | 0.006 | 1.527 | 32 | 47 | 619 | positive regulation of cellular component organization |
| GO:2000026 | 0.006 | 1.375 | 63 | 83 | 1215 | regulation of multicellular organismal development |
| GO:0007442 | 0.007 | 10.941 | 0 | 3 | 8 | hindgut morphogenesis |
| GO:0014820 | 0.007 | 10.941 | 0 | 3 | 8 | tonic smooth muscle contraction |
| GO:0034501 | 0.007 | 10.941 | 0 | 3 | 8 | protein localization to kinetochore |
| GO:0043097 | 0.007 | 10.941 | 0 | 3 | 8 | pyrimidine nucleoside salvage |
| GO:0061525 | 0.007 | 10.941 | 0 | 3 | 8 | hindgut development |
| GO:0050000 | 0.007 | 4.807 | 1 | 5 | 24 | chromosome localization |
| GO:0060612 | 0.007 | 4.807 | 1 | 5 | 24 | adipose tissue development |
| GO:0071398 | 0.007 | 4.807 | 1 | 5 | 24 | cellular response to fatty acid |
| GO:0002690 | 0.007 | 3.183 | 3 | 8 | 54 | positive regulation of leukocyte chemotaxis |
| GO:0031109 | 0.007 | 3.115 | 3 | 8 | 55 | microtubule polymerization or depolymerization |
| GO:0030512 | 0.007 | 2.892 | 3 | 9 | 66 | negative regulation of transforming growth factor beta receptor signaling pathway |
| GO:0000075 | 0.007 | 1.905 | 12 | 21 | 224 | cell cycle checkpoint |
| GO:0001568 | 0.007 | 1.638 | 22 | 34 | 418 | blood vessel development |
| GO:0048667 | 0.007 | 1.593 | 25 | 38 | 480 | cell morphogenesis involved in neuron differentiation |
| GO:0043408 | 0.007 | 1.589 | 25 | 38 | 481 | regulation of MAPK cascade |
| GO:0040012 | 0.007 | 1.561 | 28 | 41 | 528 | regulation of locomotion |
| GO:1902533 | 0.007 | 1.506 | 33 | 48 | 640 | positive regulation of intracellular signal transduction |
| GO:0072521 | 0.007 | 1.423 | 47 | 64 | 903 | purine-containing compound metabolic process |
| GO:0065008 | 0.007 | 1.257 | 143 | 170 | 2750 | regulation of biological quality |
| GO:0023052 | 0.007 | 1.217 | 250 | 281 | 4784 | signaling |
| GO:0044700 | 0.007 | 1.217 | 250 | 281 | 4784 | single organism signaling |
| GO:0006145 | 0.008 | 36.431 | 0 | 2 | 3 | purine nucleobase catabolic process |
| GO:0006434 | 0.008 | 36.431 | 0 | 2 | 3 | seryl-tRNA aminoacylation |
| GO:0009176 | 0.008 | 36.431 | 0 | 2 | 3 | pyrimidine deoxyribonucleoside monophosphate metabolic process |
| GO:0010727 | 0.008 | 36.431 | 0 | 2 | 3 | negative regulation of hydrogen peroxide metabolic process |
| GO:0019087 | 0.008 | 36.431 | 0 | 2 | 3 | transformation of host cell by virus |
| GO:0034441 | 0.008 | 36.431 | 0 | 2 | 3 | plasma lipoprotein particle oxidation |
| GO:0035022 | 0.008 | 36.431 | 0 | 2 | 3 | positive regulation of Rac protein signal transduction |
| GO:0043146 | 0.008 | 36.431 | 0 | 2 | 3 | spindle stabilization |
| GO:0043932 | 0.008 | 36.431 | 0 | 2 | 3 | ossification involved in bone remodeling |
| GO:0046078 | 0.008 | 36.431 | 0 | 2 | 3 | dUMP metabolic process |
| GO:0050955 | 0.008 | 36.431 | 0 | 2 | 3 | thermoception |
| GO:0051984 | 0.008 | 36.431 | 0 | 2 | 3 | positive regulation of chromosome segregation |
| GO:0052251 | 0.008 | 36.431 | 0 | 2 | 3 | induction by organism of defense response of other organism involved in symbiotic interaction |
| GO:1990164 | 0.008 | 36.431 | 0 | 2 | 3 | histone H2A phosphorylation |
| GO:0007143 | 0.008 | 6.084 | 1 | 4 | 16 | female meiosis |
| GO:0034502 | 0.008 | 4.566 | 1 | 5 | 25 | protein localization to chromosome |
| GO:1901655 | 0.008 | 4.566 | 1 | 5 | 25 | cellular response to ketone |
| GO:0014909 | 0.008 | 3.917 | 2 | 6 | 34 | smooth muscle cell migration |
| GO:0045599 | 0.008 | 3.917 | 2 | 6 | 34 | negative regulation of fat cell differentiation |
| GO:0032655 | 0.008 | 3.369 | 2 | 7 | 45 | regulation of interleukin-12 production |
| GO:0060113 | 0.008 | 3.369 | 2 | 7 | 45 | inner ear receptor cell differentiation |
| GO:0006338 | 0.008 | 2.367 | 5 | 12 | 105 | chromatin remodeling |
| GO:0033002 | 0.008 | 2.294 | 6 | 13 | 117 | muscle cell proliferation |
| GO:0031960 | 0.008 | 2.216 | 7 | 14 | 130 | response to corticosteroid |
| GO:0060541 | 0.008 | 1.982 | 10 | 18 | 185 | respiratory system development |
| GO:0044344 | 0.008 | 1.931 | 10 | 19 | 200 | cellular response to fibroblast growth factor stimulus |
| GO:0000082 | 0.008 | 1.877 | 12 | 21 | 227 | G1/S transition of mitotic cell cycle |
| GO:0051272 | 0.008 | 1.799 | 14 | 24 | 270 | positive regulation of cellular component movement |
| GO:0032870 | 0.008 | 1.625 | 22 | 34 | 421 | cellular response to hormone stimulus |
| GO:0035295 | 0.008 | 1.611 | 23 | 35 | 437 | tube development |
| GO:0007169 | 0.008 | 1.487 | 34 | 49 | 661 | transmembrane receptor protein tyrosine kinase signaling pathway |
| GO:0042127 | 0.008 | 1.359 | 64 | 83 | 1227 | regulation of cell proliferation |
| GO:0007154 | 0.008 | 1.211 | 253 | 284 | 4854 | cell communication |
| GO:0051716 | 0.008 | 1.207 | 274 | 305 | 5250 | cellular response to stimulus |
| GO:0001886 | 0.009 | 9.117 | 0 | 3 | 9 | endothelial cell morphogenesis |
| GO:0008655 | 0.009 | 9.117 | 0 | 3 | 9 | pyrimidine-containing compound salvage |
| GO:0046113 | 0.009 | 9.117 | 0 | 3 | 9 | nucleobase catabolic process |
| GO:0051988 | 0.009 | 9.117 | 0 | 3 | 9 | regulation of attachment of spindle microtubules to kinetochore |
| GO:0060346 | 0.009 | 9.117 | 0 | 3 | 9 | bone trabecula formation |
| GO:0060638 | 0.009 | 9.117 | 0 | 3 | 9 | mesenchymal-epithelial cell signaling |
| GO:0060603 | 0.009 | 3.782 | 2 | 6 | 35 | mammary gland duct morphogenesis |
| GO:0032615 | 0.009 | 3.283 | 2 | 7 | 46 | interleukin-12 production |
| GO:0051261 | 0.009 | 2.747 | 4 | 9 | 69 | protein depolymerization |
| GO:0051897 | 0.009 | 2.747 | 4 | 9 | 69 | positive regulation of protein kinase B signaling |
| GO:0045766 | 0.009 | 2.459 | 5 | 11 | 93 | positive regulation of angiogenesis |
| GO:0030278 | 0.009 | 2.056 | 8 | 16 | 159 | regulation of ossification |
| GO:0010948 | 0.009 | 1.859 | 12 | 21 | 229 | negative regulation of cell cycle process |
| GO:0061564 | 0.009 | 1.571 | 25 | 37 | 473 | axon development |
| GO:0040008 | 0.009 | 1.532 | 28 | 41 | 537 | regulation of growth |
| GO:0051726 | 0.009 | 1.451 | 38 | 53 | 732 | regulation of cell cycle |
| GO:0090101 | 0.01 | 2.429 | 5 | 11 | 94 | negative regulation of transmembrane receptor protein serine/threonine kinase signaling pathway |
| GO:0051384 | 0.01 | 2.229 | 6 | 13 | 120 | response to glucocorticoid |
| GO:0070371 | 0.01 | 2.087 | 8 | 15 | 147 | ERK1 and ERK2 cascade |
| GO:0001503 | 0.01 | 1.739 | 15 | 25 | 290 | ossification |

**Supplemental Table 9: List of enriched pathways**

| **GSEA enriched pathways enriched for Up-regulated genes** |  |  |  |  |  |
| --- | --- | --- | --- | --- | --- |
| **NAME** | **SIZE** | **ES** | **NES** | **NOM p-val** | **FDR q-val** |
| REACTOME_INTERFERON_SIGNALING | 54 | 0.7131298 | 3.7616508 | 0 | 0 |
| REACTOME_INTERFERON_ALPHA_BETA_SIGNALING | 32 | 0.8088969 | 3.6449168 | 0 | 0 |
| REACTOME_CYTOKINE_SIGNALING_IN_IMMUNE_SYSTEM | 71 | 0.6349219 | 3.6115007 | 0 | 0 |
| REACTOME_IMMUNE_SYSTEM | 146 | 0.4842456 | 3.186971 | 0 | 0 |
| KEGG_SYSTEMIC_LUPUS_ERYTHEMATOSUS | 28 | 0.66949093 | 2.9796486 | 0 | 0 |
| REACTOME_ANTIVIRAL_MECHANISM_BY_IFN_STIMULATED_GENES | 18 | 0.75407827 | 2.8997355 | 0 | 0 |
| REACTOME_RNA_POL_I_PROMOTER_OPENING | 18 | 0.7645554 | 2.8808575 | 0 | 0 |
| REACTOME_INTERFERON_GAMMA_SIGNALING | 26 | 0.6644058 | 2.8753316 | 0 | 0 |
| REACTOME_RNA_POL_I_TRANSCRIPTION | 18 | 0.7645554 | 2.863553 | 0 | 0 |
| REACTOME_MEIOTIC_SYNAPSIS | 18 | 0.75087416 | 2.818658 | 0 | 0 |
| REACTOME_MEIOTIC_RECOMBINATION | 20 | 0.70832014 | 2.7954347 | 0 | 0 |
| REACTOME_RNA_POL_I_RNA_POL_III_AND_MITOCHONDRIAL_TRANSCRIPTION | 21 | 0.6971262 | 2.791112 | 0 | 0 |
| REACTOME_MEIOSIS | 23 | 0.66502327 | 2.7176166 | 0 | 0 |
| REACTOME_PACKAGING_OF_TELOMERE_ENDS | 16 | 0.7746413 | 2.7024577 | 0 | 0 |
| REACTOME_TELOMERE_MAINTENANCE | 19 | 0.69721264 | 2.6875367 | 0 | 0 |
| REACTOME_AMYLOIDS | 22 | 0.6716169 | 2.6801531 | 0 | 0 |
| REACTOME_TRANSCRIPTION | 24 | 0.6415986 | 2.672456 | 0 | 0 |
| KEGG_TOLL_LIKE_RECEPTOR_SIGNALING_PATHWAY | 15 | 0.7296449 | 2.5620263 | 0 | 0 |
| REACTOME_DEPOSITION_OF_NEW_CENPA_CONTAINING_NUCLEOSOMES_AT_THE_CENTROMERE | 21 | 0.6421733 | 2.5184078 | 0 | 5.03E-05 |
| REACTOME_CHROMOSOME_MAINTENANCE | 27 | 0.55690956 | 2.4461884 | 0 | 1.33E-04 |
| REACTOME_INNATE_IMMUNE_SYSTEM | 39 | 0.4956323 | 2.4131095 | 0 | 1.70E-04 |
| KEGG_ANTIGEN_PROCESSING_AND_PRESENTATION | 16 | 0.64886266 | 2.330426 | 0 | 3.35E-04 |
| REACTOME_CLASS_A1_RHODOPSIN_LIKE_RECEPTORS | 23 | 0.5127845 | 2.1116374 | 0 | 0.002815206 |
| REACTOME_PEPTIDE_LIGAND_BINDING_RECEPTORS | 18 | 0.59381586 | 2.1808069 | 0.001533742 | 0.001398274 |
| KEGG_CYTOKINE_CYTOKINE_RECEPTOR_INTERACTION | 35 | 0.48693198 | 2.285642 | 0.002797203 | 5.22E-04 |
| REACTOME_G_ALPHA_I_SIGNALLING_EVENTS | 20 | 0.5124364 | 2.023561 | 0.003058104 | 0.006148413 |
| KEGG_CHEMOKINE_SIGNALING_PATHWAY | 23 | 0.49434066 | 2.0252922 | 0.004392386 | 0.006278829 |
| KEGG_LYSOSOME | 23 | 0.47570294 | 1.9267397 | 0.004716981 | 0.013415375 |
| REACTOME_GPCR_LIGAND_BINDING | 33 | 0.3943393 | 1.8186182 | 0.011347517 | 0.027268864 |
| KEGG_NATURAL_KILLER_CELL_MEDIATED_CYTOTOXICITY | 19 | 0.48095715 | 1.8478309 | 0.012718601 | 0.022847803 |
| KEGG_CELL_ADHESION_MOLECULES_CAMS | 35 | 0.3321812 | 1.5650082 | 0.054285713 | 0.116300985 |
| REACTOME_ADAPTIVE_IMMUNE_SYSTEM | 69 | 0.26339754 | 1.4869835 | 0.06426735 | 0.16437249 |
| REACTOME_GPCR_DOWNSTREAM_SIGNALING | 41 | 0.3075744 | 1.4841685 | 0.08033241 | 0.16134441 |
| PID_CXCR4_PATHWAY | 20 | 0.38332626 | 1.483987 | 0.092920355 | 0.1567628 |
| REACTOME_SIGNALING_BY_GPCR | 51 | 0.27477103 | 1.4242573 | 0.10123119 | 0.2003542 |

**GSEA enriched pathways enriched for down-regulated genes**

| **NAME** | **SIZE** | **ES** | **NES** | **NOM p-val** | **FDR q-val** |
| --- | --- | --- | --- | --- | --- |
| REACTOME_CELL_CYCLE_MITOTIC | 50 | -0.4548963 | -3.0784822 | 0 | 0 |
| REACTOME_DNA_REPLICATION | 32 | -0.5146689 | -2.9132993 | 0 | 0 |
| REACTOME_MITOTIC_M_M_G1_PHASES | 26 | -0.5546116 | -2.8787684 | 0 | 0 |
| REACTOME_MITOTIC_PROMETAPHASE | 15 | -0.61606574 | -2.547545 | 0 | 3.01E-04 |
| PID_FOXM1PATHWAY | 15 | -0.49651012 | -2.0478098 | 0.002557545 | 0.020803189 |
| REACTOME_G1_S_TRANSITION | 16 | -0.40012616 | -1.7148101 | 0.0259366 | 0.12146987 |
| REACTOME_G_ALPHA_Q_SIGNALLING_EVENTS | 17 | -0.38317028 | -1.6804874 | 0.038251366 | 0.13236023 |
| REACTOME_GASTRIN_CREB_SIGNALLING_PATHWAY_VIA_PKC_AND_MAPK | 19 | -0.36697832 | -1.7151573 | 0.020231213 | 0.14171486 |
| REACTOME_MHC_CLASS_II_ANTIGEN_PRESENTATION | 15 | -0.3856215 | -1.6256145 | 0.04945055 | 0.15822655 |
| REACTOME_MITOTIC_G1_G1_S_PHASES | 19 | -0.34900716 | -1.5770079 | 0.037249282 | 0.18427815 |
| PID_INTEGRIN1_PATHWAY | 18 | -0.34438127 | -1.5387758 | 0.04178273 | 0.20317405 |
